# Glucose competition between endothelial cells in the blood-spinal cord barrier and infiltrating regulatory T cells is linked to sleep restriction-induced hyperalgesia

**DOI:** 10.1186/s12916-024-03413-z

**Published:** 2024-05-07

**Authors:** Yulin Huang, Rui Xu, Qi Liu, Xiao Zhang, Yanting Mao, Yan Yang, Xiaoping Gu, Yue Liu, Zhengliang Ma

**Affiliations:** Department of Anesthesiology, Nanjing Drum Tower Hospital, The Affiliated Hospital Medical School, Nanjing University, No. 321 of Zhongshan Road, Nanjing, 210008 China

**Keywords:** Sleep restriction, Hyperalgesia, Blood-spinal barrier, Endothelial cells, Glycolysis, Tregs

## Abstract

**Background:**

Sleep loss is a common public health problem that causes hyperalgesia, especially that after surgery, which reduces the quality of life seriously.

**Methods:**

The 48-h sleep restriction (SR) mouse model was created using restriction chambers. *In vivo* imaging, transmission electron microscopy (TEM), immunofluorescence staining and Western blot were performed to detect the status of the blood-spinal cord barrier (BSCB). Paw withdrawal mechanical threshold (PWMT) was measured to track mouse pain behavior. The role of infiltrating regulatory T cells (Tregs) and endothelial cells (ECs) in mouse glycolysis and BSCB damage were analyzed using flow cytometry, Western blot, CCK-8 assay, colorimetric method and lactate administration.

**Results:**

The 48-h SR made mice in sleep disruption status and caused an acute damage to the BSCB, resulting in hyperalgesia and neuroinflammation in the spinal cord. In SR mice, the levels of glycolysis and glycolysis enzymes of ECs in the BSCB were found significantly decreased [CON group vs. SR group: CD31^+^Glut1^+^ cells: *p* < 0.001], which could cause dysfunction of ECs and this was confirmed *in vitro*. Increased numbers of infiltrating T cells [*p* < 0.0001] and Treg population [*p* < 0.05] were detected in the mouse spinal cord after 48-h SR. In the co-cultured system of ECs and Tregs *in vitro*, the competition of Tregs for glucose resulted in the glycolysis disorder of ECs [Glut1: *p* < 0.01, ENO1: *p* < 0.05, LDHα: *p* < 0.05; complete tubular structures formed: *p* < 0.0001; CCK8 assay: *p* < 0.001 on 24h, *p* < 0.0001 on 48h; glycolysis level: *p* < 0.0001]. An administration of sodium lactate partially rescued the function of ECs and relieved SR-induced hyperalgesia. Furthermore, the mTOR signaling pathway was excessively activated in ECs after SR *in vivo* and those under the inhibition of glycolysis or co-cultured with Tregs *in vitro*.

**Conclusions:**

Affected by glycolysis disorders of ECs due to glucose competition with infiltrating Tregs through regulating the mTOR signaling pathway, hyperalgesia induced by 48-h SR is attributed to neuroinflammation and damages to the barriers, which can be relieved by lactate supplementation.

**Supplementary Information:**

The online version contains supplementary material available at 10.1186/s12916-024-03413-z.

## Background

Sleep loss is a public health issue of concern. Numerous health problems result from insufficient sleep. It not only significantly increases the incidence of cancers, sudden death and cardiovascular diseases, but also impairs learning and cognitive abilities and causes negative emotions [1]. Hyperalgesia is a severe outcome of sleep loss, which particularly occurs after surgery [2, 3]. Postoperative hyperalgesia remarkably weakens the analgesic effect of opioids and induces restlessness, high stress reactions, unpleasant emotional and psychological reactions and even more severe complications [4, 5]. Identifying the mechanisms underlying sleep loss induced postoperative hyperalgesia, is therefore of great significance.

A dynamic control of vascular integrity and permeability is essential for normal organ function. The occurrence and development of pain are closely related to the destruction of the blood-brain barrier (BBB) and blood-spinal cord barrier (BSCB). An instantaneous permeability of the BBB and BSCB has been illustrated in pain models of inflammation and nerve injury, leading to nerve inflammation and pain [6–8]. Moreover, sleep loss causes damages to the barriers that possibly may be attributed to the occurrence of hyperalgesia [9, 10]. The maintenance of vascular integrity requires connections and communications between ECs and pericytes. The lack of pericytes in the central nervous system (CNS) results in the destruction of the BBB and BSCB [11]. Glucose is the main energy source supporting the CNS, and glucose transporter 1 (Glut1) mainly distributed on barrier ECs is responsible for carrying peripheral glucose to the BSCB [12–14]. Glycolysis is the essential energy-sustaining process of ECs to extensively produce lactic acid [15]. The endothelium-derived lactic acid, in turn, maintains functions of ECs, which also participates in pericyte metabolism and the maintenance of barriers [16–18]. Breakdown of the barriers and increases in the permeability have been observed in Glut1^-/-^ mice with the inhibition of lactate production in the endothelium. The potential role of SR-induced hyperalgesia in endothelial glycolysis disorders and the underlying mechanisms have been well concerned.

Peripheral immune cells used to be believed that they cannot enter the CNS, which has been found wrong with the in-depth study of the BBB and BSCB [19]. The infiltration of peripheral immune cells in the CNS stimulates the occurrence of hyperalgesia, especially that of T cells [20–23]. Glucose and glycolysis are of great significance in proinflammatory functions of immune cells, and depletion of glucose in the pathological microenvironment triggers defective immune responses [24]. Mammalian target of rapamycin (mTOR) signaling pathway is a classic signaling transduction involved in glucose metabolism and regeneration of damaged neural tissues in the chronic phase [25–28]. Park et al. [26] found the activated mTOR signaling pathway in blood vessels surrounding the injured spinal cord and in oxygen and glucose deprivation (OGD)/reperfusion-induced ECs. We thereafter hypothesized that T cells during their infiltration into the spinal cord, may compete with ECs for glucose to cause glycolysis disorder after SR through activating the mTOR signaling pathway. We analyzed hyperalgesia in SR mice caused by damaging the BSCB and the underlying mechanism, and provided theoretical references for the management of postoperative pain.

## Methods

### Animals

Animal experiments were performed under a project license (No. 2020AE01025) granted by the Institutional Ethics Board of Nanjing Drum Tower Hospital, the Affiliated Hospital of Nanjing University Medical School, in compliance with the ARRIVE guidelines and in accordance with the U.K. Animals (Scientific Procedures) Act, 1986 and associated guidelines, EU Directive 2010/63/EU for animal experiments, or the National Institutes of Health guide for the care and use of Laboratory animals (NIH Publications No. 8023, revised 1978). Considering the stability of weight, male C57BL/6 mice (*n*=170, weight 20–25 g; 6 weeks old) were housed in a specific pathogen-free (SPF) environment with the constant temperature (22±2°C), constant humidity (55±5%) and light dark cycle for 12:12 h, and given free accesses to water and food.

### Sleep restriction model

Mice were randomly divided into control (CON group) and SR group. A 48-h SR model was used as previously described [27, 28]. For continuous SR, each mouse was placed in a restriction chamber (XR-XS108, Xinruan Information Technology Co. Ltd., Shanghai, China), in which a rotating bar with a short distance above the cage floor was kept under constant but gently motive to limit sleep for 48 h. The bar was programmed to move at a speed of seven in the moderate mode and alternate between clockwise and counter-clockwise rotations. Non-deprived mice were placed in a similar sized chamber without bar rotation. An equal amount of food was provided to mice of both groups. Mouse weight was recorded during the process of 48-h sleep restriction.

### Incisional surgery

Postoperative pain after 48-h SR was performed as previously described [29]. Briefly, mice were anesthetised with sevoflurane delivered via a small nose cone. The plantar aspect of the right hind paw was prepared in a sterile manner using a 10% povidone–iodine solution, and the foot was placed through a hole in a sterile drape. A 0.5-cm longitudinal incision was made with a surgical blade through the skin and fascia of the plantar region, starting 0.5 cm from the proximal edge of the heel and extending towards the toes. The plantar muscles were exposed, elevated, and incised longitudinally. After haemostasis was achieved with gentle pressure, the skin was sutured using two mattress sutures of 5-0 nylon. The wound site was covered with aureomycin ointment.

### Sodium lactate treatment

SR mice with or without undergoing incisional surgery were divided into phosphate-buffered saline group (PBS; SR + PBS) and sodium lactate group (SR + NaLA). Mice in the SR + NaLA group were administered 2 g/kg sodium lactate dissolved in PBS (Sigma-Aldrich, Darmstadt, Germany) by intraperitoneal injections 5 days a week from 30 minutes before SR to 2 weeks later at a fixed time point. Sodium lactate can cross the blood-spinal barrier and a short period of lactic acid build-up in the body caused transient muscle weakness in the mice, which returned to normal after 30 min. So the SD and PWMT were all done 30 min after lactate administration.

### Sleep monitoring

Mice were placed in a restriction chamber connected to an electroencephalography/electromyography (EEG/EMG) system (Blackrock Microsystems Cereplex Direct, Utah, USA) to monitor sleep. The matched head-mounted preamplifiers were implanted surgically. For EEG/EMG monitoring, mice were anesthetized with 2% isoflurane using animal anaesthesia machines (91805060; Midmark VMR, OH, USA) and maintained at the anaesthetic flow of 0.2 L/min. Then, mice were mounted onto a stereotactic apparatus (David Kopf Instrument, CA, USA) for surgery. A 1.5 cm rostral-caudal incision was made approximately 3.5 cm anterior to the bregma, and the EEG/EMG head mount was adhered to the surface of the dry skull using glass ionomer cement (Shangchi Dental Materials, Jiangsu, China). Screws (S-BORUI 304 stainless steel fasteners M1, Siborui, Guangdong, China) were drilled into the skull at the corresponding holes in the head mount for hippocampal and cortical EEG recordings. Small pockets were made in the nuchal muscles to insert the EMG wires. The skin around the head mount was sutured, and mice were allowed to recover for at least a week. The implanted mice were tethered to the EEG/EMG system in a restriction chamber and acclimatized for 24 h before recording. Recordings were acquired under standard conditions for 48 h without bar rotation, followed by another 48 h with it. Sleep recordings were scored in 10-s epochs over 48 h of normal sleep and sleep restriction using the union stage sleep analysis software.

### Paw withdrawal mechanical threshold (PWMT)

Mechanical allodynia was induced using a series of von Frey filaments (Stoelting, IL, USA) [30–32]. Briefly, mice were placed into individual transparent plexiglass compartments (10×10×15 cm) on a metal mesh floor (graticule: 0.5×0.5 cm). After acclimatization for at least 30 min, von Frey filaments (0.16, 0.4, 0.6, 1.0, 1.4, and 2.0 g) were vertically poured onto the plantar surface of the right hind paw and held in place for 6–8 s, which was repeated five times. The lowest stimulus strength that caused brisk withdrawal of the paw or paw flinching, which produced three or more positive responses in a total of five times, was regarded as the PWMT.

### Imaging examinations

After SR or treatment, mice in CON group, SR group, SR + PBS group and SR + NaLA group were injected intraperitoneally with fluorescein sodium (NaFlu, 10%, 4.0 mL/kg. F6377; Sigma-Aldrich, Darmstadt, Germany). Mice were sacrificed 24 hours later, and their brain and spinal tissues were completely removed and placed on ice. The quantified emission light intensity was represented as the total radiant efficiency (TRE) to detect the distribution of NaFlu in an Optical *In Vivo* Imaging System (IVIS® Lumia series III, PerkinElmer, MA, USA). The brain and spinal tissues were imaged at an excitation wavelength of 440 nm and an emission wavelength of 520 nm.

### Transmission electron microscopy (TEM)

After SR and sodium lactate treatment, mice in CON group, SR group, SR + PBS group and SR + NaLA group were sacrificed under overdose isoflurane. The entire lumbosacral enlargement was quickly removed, prepared for tissue sections, and immediately soaked in the fixation liquid (Vazyme, Jiangsu, China). After embedding in epoxy resin, tissue sections were cut into 60-mm slides and stained with uranyl acetate and lead citrate. Images were captured using a Hitachi 7100 electron microscope (Hitachi, Tokyo, Japan).

### Immunofluorescence staining

The lumbosacral enlargement was collected in mice of CON group and SR group, fixed in 4% paraformaldehyde, and dehydrated in 30% sucrose. The third to fifth lumbar vertebrae (L3–L5) were transversely cut into 25-μm-thick sections and mounted on glass slides. Sections were blocked and then incubated with primary antibodies against Iba-1 (1:700, Wako, Tokyo, Japan) and GFAP (1:500, Abcam, Cambridge, UK) or CD31 (1:500, Abcam, Cambridge, UK) overnight at 4°C. After washing with PBS, the sections were incubated with Alexa 488-conjugated goat anti-rabbit antibody (1:1000, Thermo Fisher Scientific, MA, USA) and Alexa 594-conjugated goat anti-mouse antibody (1:1000, Thermo Fisher Scientific, MA, USA) in the dark. Three spinal cord sections per animal were used for statistical analysis. The fluorescence intensities of Iba1, GFAP, and CD31 were measured using ImageJ software (NIH, Bethesda, MD, USA).

### Flow cytometry

Mouse spinal samples were collected on the first day after SR. They were minced into small pieces and digested in serum-free RPMI 1640 medium containing DNase I (50 U/ml), hyaluronidase (100 μg/ml), collagenase type IV (1 mg/ml), and L-glutamine (2 μM) (all from Sigma, MO, USA) for 0.5 hour at room temperature (RT) with a low rotation. Single-cell suspension was obtained by filtering the digested tissues through 40-μm cell strainers (BD Falcon, San Jose, USA). For cell surface staining, aliquots of single-cell suspensions (1.0×10^6^) were incubated with fluorophore-conjugated monoclonal antibodies at RT in the dark (FITC-anti-CD31, AF647-anti-Glut1, Alexa Fluor 488-anti-CD3, PE-cy7-anti-CD4, APC-anti-CD25, PerCP-anti-CD45, FITC-anti-CD11b; BioLegend, CA, USA). For intracellular staining, cells were incubated for 4 hours in RPMI 1640 medium, phorbol 12-myristate 13-acetate (50 ng/mL), ionomycin (1 μg/mL), and brefeldin 5% CO_2_ atmosphere at 37°C. Cells were washed with PBS, fixed, permeabilized, and stained with PE-anti-IL-17A (BioLegend, CA, USA) or AF647-anti-GFAP (without stimulation) according to the manufacturer’s protocol. For intranuclear transcription factor detection, the cells were washed, fixed, permeabilized, and stained with PE-anti-Foxp3 (BD Biosciences, CA, USA) according to the manufacturer’s protocol. Tregs were defined as CD4^+^CD25^+^ cells. Astrocytes were defined as GFAP^+^ cells. Microglia were defined as CD45^int^CD11b^hi^ cells. Fluorescence data were collected on a FACS Aria II (BD Biosciences, CA, USA) and analysed using FlowJo software (Tree Star, OR, USA).

### Tregs sorting and cell culture

CD4^+^ Tregs were sorted from spleen cells of SR mice using Dynabeads™ FlowComp™ Mouse CD4^+^CD25^+^ Treg Cells Kit (Invitrogen, CA, USA) according to the manufacturer’s protocol. The purity of the sorted cells was validated by flow cytometry.

The human endothelial cell line HUVEC was purchased from ATCC (American Type Culture Collection, VA, USA) and cultured in DMEM or DMEM low glucose liquid (both from Gibco, CA, USA) supplemented with 10% FBS at 37°C in 5% CO_2_.

### Co-culture experiments

CD4^+^ Tregs (6.0×10^4^/ml) sorted from spleen cells of SR mice were cultured with HUVECs (2.0×10^5^/ml) separated in a Transwell chamber with DMEM in 24-well plates at 37°C in 5% CO_2_. After 2 days, the HUVECs were harvested for subsequent experiments.

### Tube formation assay

A 96-well plate was coated with 50 μL of Matrigel TM (BD Biosciences, USA) and kept at 37°C for 2 hours. Then, 2.0×10^4^ HUVECs were suspended in 100 μL of conditioned medium and applied to the pre-coated 96-well plate. After incubation at 37°C for another 24 hours, images were taken under a microscope and the tubular structures formed in the Matrigel were counted in five random fields.

### CCK-8 assay

Cell proliferation was assessed using the Cell Counting Kit-8 (CCK-8) (Vazyme, Jiangsu, China) according to the manufacturer’s instructions. Briefly, cells in different culture conditions were plated into 96-well plates at 1.0×10^4^ cells/well with 100 μL of complete medium. At the indicated time points (12, 24 and 48 hours), cells were incubated with 100 μL of complete medium plus 10 μL of CCK-8 reagent at 37°C for 2 hours. Absorbance was measured using a microplate reader (Bio-Rad, CA, USA) at a wavelength of 450 nm.

### Glycolysis level detection

HUVECs were collected, washed and plated in 96-well plates at 5.0×10^3^ cells/well with 120 μL of RPMI 1640 medium containing 2% FBS to starve cells overnight. Cell supernatant was collected from each well the next morning. Each sample was analysed in triplicate. A Glycolysis Cell-Based Assay Kit (Cayman, MI, USA) was used to detect glycolysis levels according to the manufacturer’s instructions.

### Western blotting

Tissues and cells were harvested and prepared for the whole-cell lysates using ice-cold RIPA lysis buffer containing a protease inhibitor cocktail and lysates. The rabbit monoclonal antibodies used for Western blotting were anti-Glut1, anti-lactate dehydrogenase α (LDHα), anti-Enolase 1 (ENO1), anti-ZO-1, anti-Occludin, anti-Claudin-5 (all from Affinity Bioscience, Jiangsu, China), anti-phospho-mTOR, anti-phospho-p70S6K, anti-phospho-AMPK, anti-phospho-4E-BP1, and anti-β-actin antibodies (Cell Signaling Technology, BO, USA). Goat anti-rabbit IgG (Beyotime, Shanghai, China) was used as the secondary antibody. Western blotting was performed using a chemiluminescent substrate (Vazyme, Jiangsu, China). Images were captured using a cooled charge coupled device (CCD; Tanon, Shanghai, China). Each blot was performed in triplicate. Quantitative densitometry analysis was performed using the ImageJ software (NIH, Bethesda, MD, USA).

### Statistical analysis

Statistical analysis was performed using SPSS (Statistical Package for the Social Science) 22.0 software (IBM Corp., Armonk, NY, USA) and all data were expressed as mean±standard deviation (SD). Changes in behavioural assessments were analysed using two-way repeated measures of variance. Differences between groups and among three or more groups were compared using the unpaired two-tailed Student’s *t* test and one-way ANOVA, respectively, followed by a post-hoc test (Bonferroni test). *P* <0.05 was considered statistically significant.

## Results

### SR induces mouse hyperalgesia and neuroinflammation

**Fig. 1:**
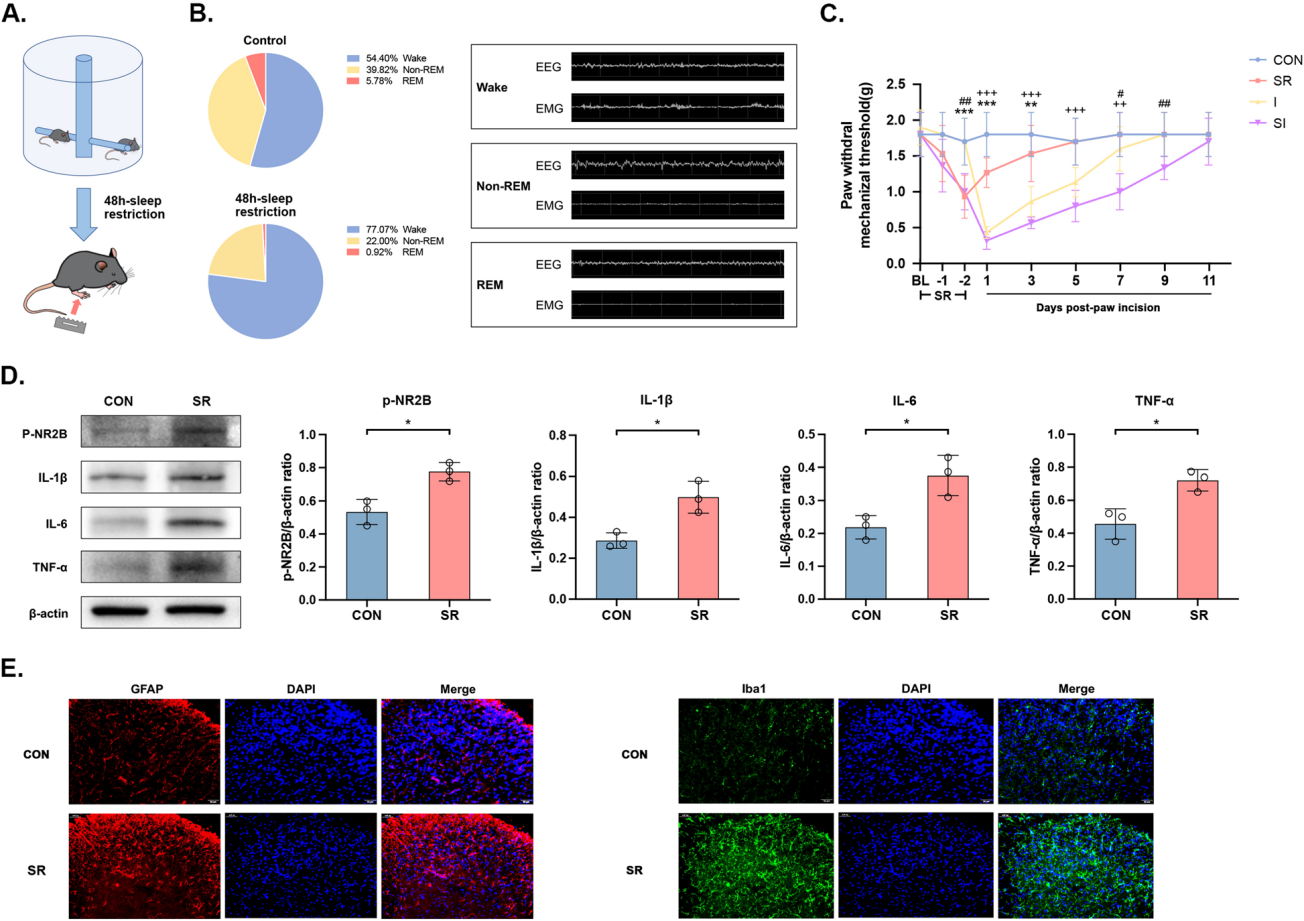
48-h SR induces hyperalgesia and neuroinflammation in mouse spinal cord. **A** Schematic diagram of creating the 48-h SR model in mice. **B** Sleep composition of mice monitored by EEG/EMG (left) and representative EEG/EMG waveforms in different sleep states of mice (right). **C** Time-dependent changes in PWMT in response to mechanical stimulation during SR (Day -2 and -1) and after incisional surgery (Day 1, 3, 5, 7, 9, and 11). *n*=8 per group. CON *vs.* SR: **p<0.01, ***p<0.001; CON *vs.* I: ^++^*p*<0.01, ^+++^*p*<0.001; I *vs.* SR+I: ^#^*p*<0.05, ^##^*p*<0.01. **D** Expression levels of p-NR2B, IL-1β, IL-6 and TNF-αdetected by Western blot (left) and their quantitative analyses (right). *n* = 3 per group. *p<0.05. **E** Immunostaining for Iba1 and GFAP in lumbar spinal dorsal horn ipsilateral to the incision on Day 1. Scale bar=50 μm. *n* = 6 per group

The 48-h SR model in mice was performed using the restriction chambers, followed by an incisional surgery (Fig. 1A). The status of sleep was verified by EEG/EMG monitoring (Fig .1B). During 48-h SR, the prolonged awakening time and shortened rapid eye movement (REM) and non-REM sleep were suggestive of the successful modelling [28]. Although the sleep was not completely abolished, mice were in a state of sleep disruption. Changes in nociceptive behaviours were then measured. Firstly, the baseline PWMT was comparable among the CON, I, SR, and SR + I groups (Fig. 1C,). Compared with that of the CON group, a significant decrease in PWMT occurred on day 2 in mice of the SR group but recovered on day 5 [CON group vs. SR group: *p* < 0.001 on Day -2, and 1, *p* < 0.01 on Day 3]. As expected, pain hypersensitivity in Group I peaked on day 1 after incisional surgery, which gradually recovered and completely disappeared on day 9 [CON group vs. I group: *p* < 0.001 on Day 1, 3 and 5, *p* < 0.01 on Day 7]. Meanwhile, mice in the SR + I group showed aggravated and prolonged postoperative pain until day 11 in mice after SR compared with that of Group I [I group vs. SR + I group: *p* < 0.01 on Day -2 and 9, *p* < 0.05 on Day 7]. These results suggested that SR induced acute hyperalgesia and prolonged post-surgical pain recovery. The phosphorylation level of the NMDA receptor NR2B subunits is found positively correlated with neuroinflammation and pro-inflammatory cytokines [33]. Here, phosphorylated NR2B (p-NR2B) and pro-inflammatory factors were significantly upregulated in SR mice [p-NR2B: *p* < 0.05, IL-1β: *p* < 0.05, IL-6: *p* < 0.05, TNF-α: *p* < 0.05] (Fig. 1D), and their levels were significantly higher in SR + I group than those of I group [p-NR2B: *p* < 0.05, IL-1β: *p* < 0.01, IL-6: *p* < 0.05, TNF-α: *p* < 0.01] (Additional file 1: Fig. S1A). Neuroinflammation is a common consequence accompanied by the activation of glial cells [34, 35]. Immunofluorescence staining and flow cytometry both revealed the activation of mouse astrocytes and microglia after 48-h SR (Fig. 1E), and increased cell numbers [astrocytes: *p* < 0.05; microglia: *p* < 0.05] (Additional file 1: Fig. S1B). Collectively, 48-h SR resulted in acute hyperalgesia and neuroinflammation as well as the prolongation of post-surgical pain recovery.

### SR induces an acute damage to the mouse BSCB

**Fig. 2:**
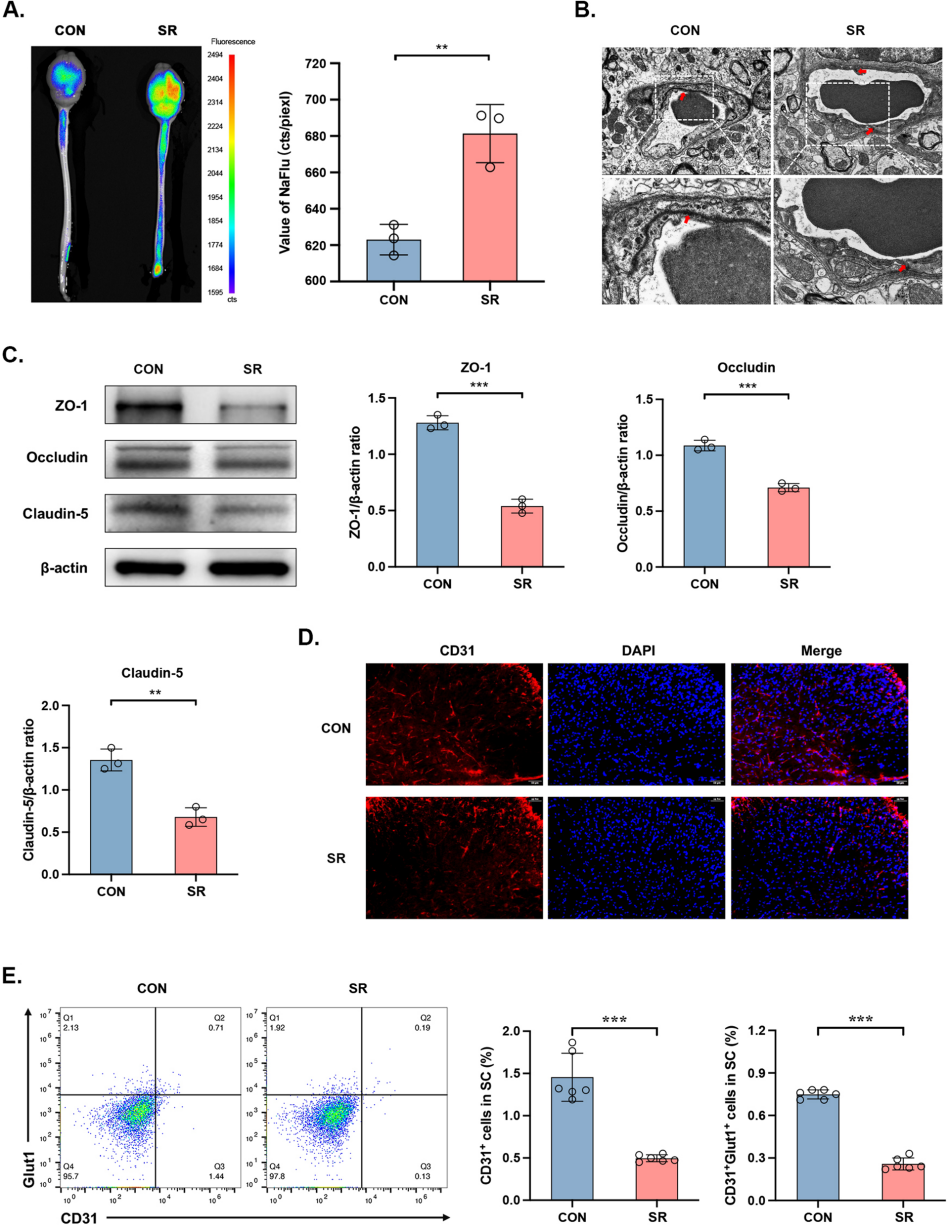
SR induces damages to the BSCB. **A** NaFlu distribution in mice (left) and the quantitative analyses (right). *n*=3 per group. **p<0.01. **B** TEM images of tight junctions in BSCB on Day 1. Scale bar=2.0 μm. *n* = 6 per group. **C** Expression levels of ZO-1, Occludin, Claudin-5 detected by Western blot (left) and their quantitative analyses (right). *n*=3 per group. **p<0.01, ***p<0.001. **D** Immunostaining for CD31 in lumbar spinal dorsal horn on Day 1. Scale bar=50 μm. *n* = 6 per group. **E** The expression level of CD31^+^Glut1^+^ cells in spinal cord (left) and the proportion (right) on Day 1. *n*=6 per group. ***p<0.001

The status of mouse BSCB and BBB was visualized by the NaFlu assay and *in vivo* imaging examination. Both the BSCB and BBB were significantly broken after 48-h of SR compared with mice in the CON group [*p* < 0.01] (Fig. 2A). TEM showed the breakdown and degradation of tight junctions of the BSCB on day 1 after SR, with a loosened and blurred structure (Fig. 2B). Downregulated biomarkers of tight junctions and ECs, and morphological destruction of ECs after 48-h SR consistently supported our findings [levels of tight junctions: ZO-1: *p* < 0.001, Occludin: *p* < 0.001, Claudin-5: *p* < 0.001; CD31^+^ cells: *p* < 0.001] (Fig. 2C-E). Glut1 is responsible for ECs to maintain the stability of BSCB [12–14, 36], which was significantly downregulated after 48-h of SR [CD31^+^Glut1^+^ cells: *p* < 0.001] (Fig. 2E). We thereafter validated the involvement of glucose metabolism of ECs in SR-induced damages of the BSCB and BBB.

### Dysfunctional ECs in low-glucose intracellular microenvironment

**Fig. 3:**
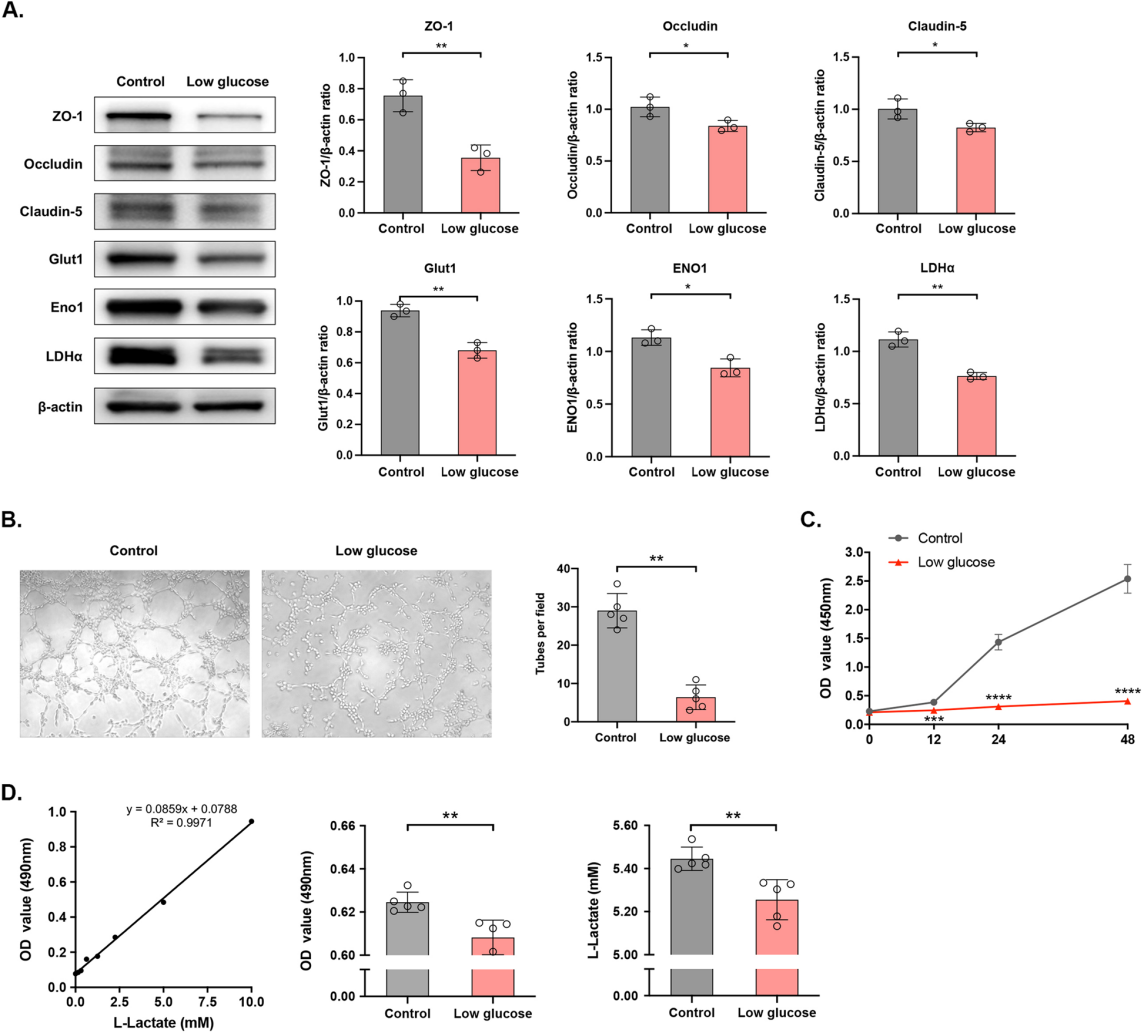
Dysfunctional ECs in low-glucose intracellular microenvironment. **A** Protein levels of ZO-1, Occludin, Claudin-5, Glut1, Eno1 and LDHα detected by Western blot (left) and their quantitative analyses (right). *n*=3 per group. *p<0.05, **p<0.01. **B** Tube formation by HUVECs in five random fields. *n*=5 per group. **p<0.01. **C** Proliferative rate of HUVECs at different time points detected by CCK-8 assay. *n*=5 per group. ***p<0.001, ****p<0.0001. **D** The standard curve of glycolysis levels in HUVECs (left), optical density (middle) and the level of L-Lactate by conversion (right). *n*=3 per group. **p<0.01

HUVECs were cultured in DMEM low glucose solution for 2 days to mimic an low-glucose intracellular microenvironment *in vitro* [37–39]. Expression levels of tight junctions and glycolytic enzymes in HUVECs cultured with DMEM low glucose solution were significantly lower than those cultured in the normal medium [ZO-1: *p* < 0.01, Occludin: *p* < 0.05, Claudin-5: *p* < 0.05, Glut1: *p* < 0.01, ENO1: *p* < 0.05, LDHα: *p* < 0.05] (Fig. 3A). Besides, the mean number of complete tubular structures formed by HUVECs, proliferative rate and glycolysis levels of HUVECs cultured in DMEM low glucose solution were significantly lower than those of negative controls [complete tubular structures formed: *p* < 0.01; CCK8 assay: *p* < 0.001 on 12h, *p* < 0.0001 on 24h and 48h; glycolysis level: *p* < 0.01] (Fig. 3B-D), suggesting the impaired biological functions of HUVECs in low-glucose intracellular microenvironment. We further analyzed the biological functions of HUVECs influenced by glycolysis, which were similarly impaired in those induced with 1 mM glycolysis inhibitor 2-deoxy-d-glucose (2-DG) [levels of proteins: ZO-1: *p* < 0.01, Occludin: *p* < 0.001, Claudin-5: *p* < 0.001, Glut1: *p* < 0.05, ENO1: *p* < 0.05, LDHα: *p* < 0.05; complete tubular structures formed: *p* < 0.0001; CCK8 assay: *p* < 0.001 on 24h and 48h; glycolysis level: *p* < 0.0001] (Additional file 2: Fig. S2A–D). It is indicated dysfunctional ECs in intracellular low-glucose microenvironment was associated with glycolytic disturbances.

### Infiltrating Tregs in the spinal cord competes with ECs for glucose

**Fig. 4:**
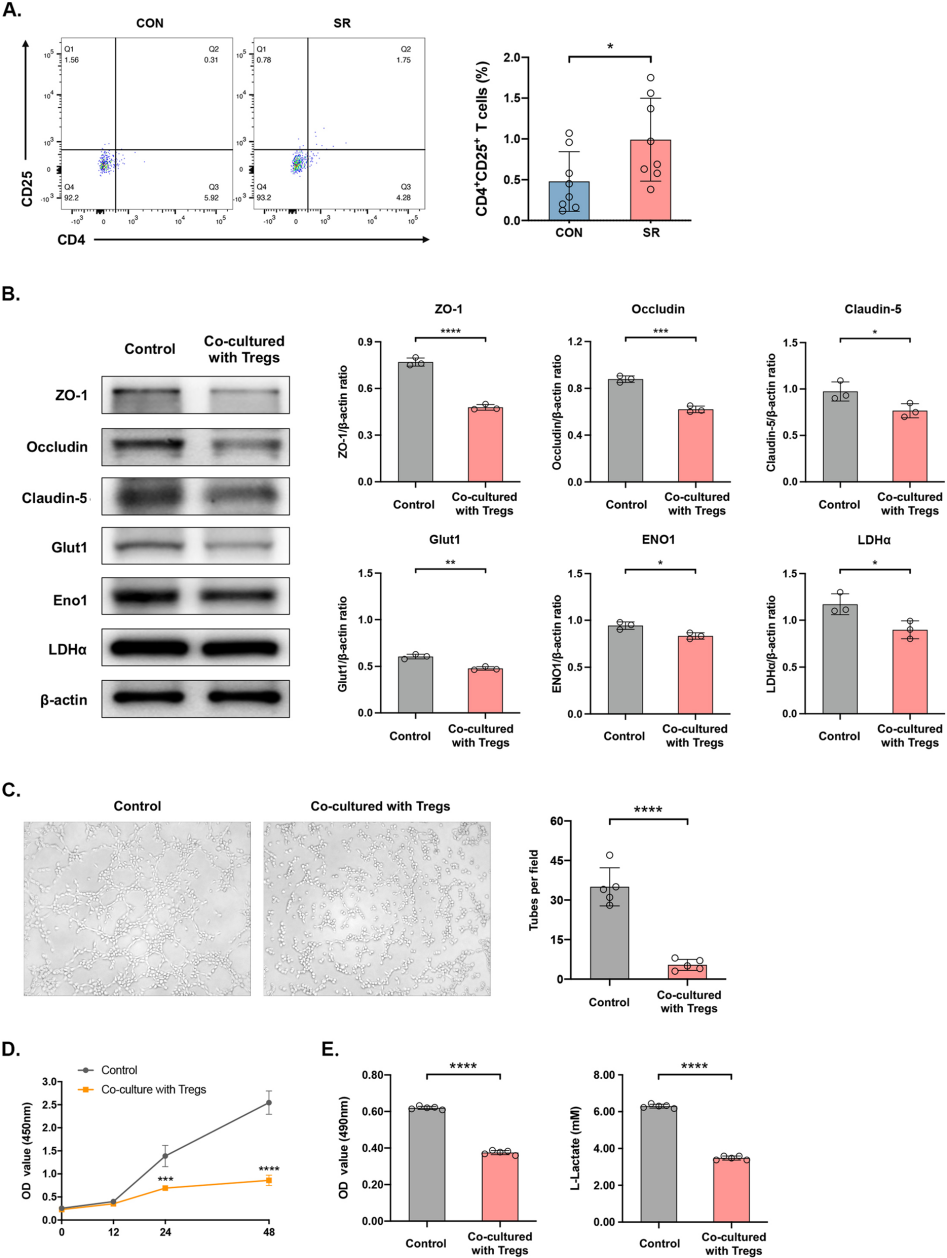
Infiltrating Tregs in the spinal cord influence functions and glycolysis levels of ECs. **A** The number of CD4^+^CD25^+^ T cells in mice spinal cord (left) and the proportion on Day 1 (right). *n*=8 per group. *p<0.05. **B** Protein levels of ZO-1, Occludin, Claudin-5, Glut1, Eno1 and LDHα detected by Western blot (left) and their quantitative analyses (right). *n*=3 per group. *p<0.05, **p<0.01, ***p<0.001, ****p<0.0001. **C** Tube formation by HUVECs in five random fields. *n*=5 per group. ****p<0.0001. **D** Proliferative rate of HUVECs at different time points detected by CCK-8 assay. *n*=5 per group. ***p<0.001, ****p<0.0001. **E** The standard curve of glycolysis levels in HUVECs (left), optical density (middle) and the level of L-Lactate by conversion (right). *n*=3 per group. ****p<0.0001

Interestingly, the number of CD3^+^ T cells in mouse spinal tissues was significantly greater in the SR group than that of the CON group [*p* < 0.0001] (Additional file 3: Fig. S3A), suggesting that T lymphocytes were infiltrated in the spinal cord after SR-induced BSCB damage. Classified by the CD3^+^ T cell subsets, a significantly greater number of CD4^+^CD25^+^ T cells but a comparable number of Th17 cells were detected in mice of SR group than those of CON group [Tregs: *p* < 0.05; Th17s: *p* > 0.05] (Fig. 4A & Additional file 3: Fig. S3A). An *in vitro* co-culture system involving Tregs sorted from mouse spleens and ECs was created to analyze the role of infiltrating Tregs in glycolytic disturbances of ECs. The purity of splenic Tregs over 85% and positive expression of Foxp3 over 98.5% were suggestive of the successful isolation of Tregs (Additional file 3: Fig. S3B&C). The expression level of Foxp3 was comparable in Tregs either co-cultured with HUVECs for 48 h or not [*p* > 0.05] (Additional file 3: Fig. S3D, E). Similarly, expression levels of tight junctions and glycolysis enzymes were significantly lower in HUVECs co-cultured with Tregs, so as the lower mean number of complete tubular structures formed by HUVECs, proliferative rate and glycolysis levels [levels of proteins: ZO-1: *p* < 0.0001, Occludin: *p* < 0.001, Claudin-5: *p* < 0.05, Glut1: *p* < 0.01, ENO1: *p* < 0.05, LDHα: *p* < 0.05; complete tubular structures formed: *p* < 0.0001; CCK8 assay: *p* < 0.001 on 24h, *p* < 0.0001 on 48h; glycolysis level: *p* < 0.0001] (Fig. 4B-E). It is concluded that Tregs may compete with ECs for glucose and cause glycolysis disturbances in ECs, leading to EC dysfunction.

### Lactate administration rescues the function of ECs and relieves SR-induced hyperalgesia

**Fig. 5:**
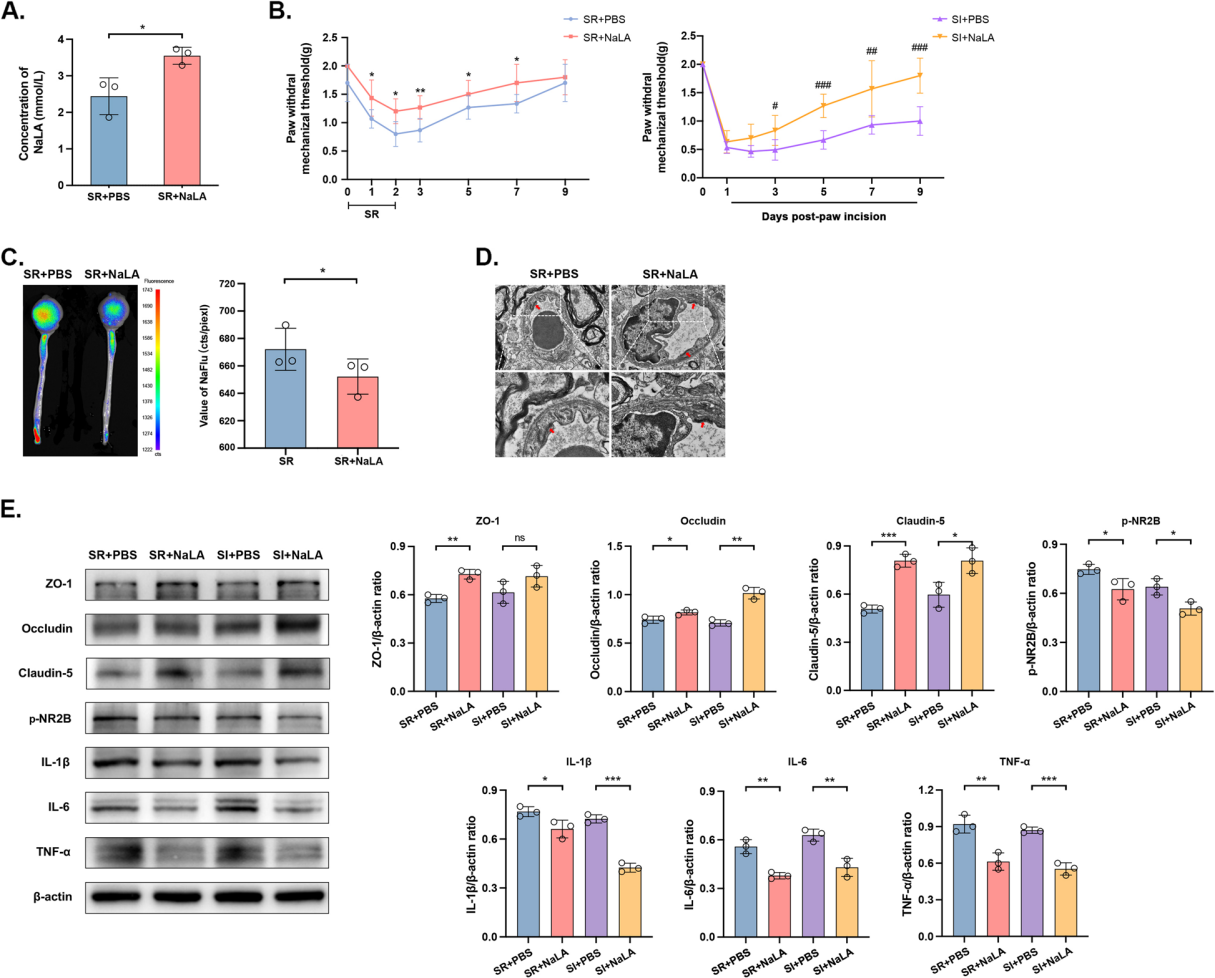
Lactate administration rescues functions of ECs and relieves SR-induced hyperalgesia. **A** Concentration of NaLA in mice spinal cord detected by colorimetry. *n*=3 per group. *p<0.05. **B** Time-dependent changes in PWMT after continuous 48-h SR (Day 0) and a week of lactate administration (Day 1, 2, 3, 5, 7, and 9) in mice (left) and those on the day of incision after SR (Day 0) and post-incision with lactate administration (Day 1, 3, 5, 7, and 9) (right). *n*=8 per group. SR + PBS *vs.* SR + NaLA: *p<0.05, **p<0.01; SI + PBS *vs.* SI + NaLA: ^#^*p*<0.05, ^##^*p*<0.01, ^###^*p*<0.001. **C** NaFlu distribution in mice taken by in vivo imaging system (left) and the quantitative analysis (right). *n*=3 per group. *p<0.05. **D** Tight junctions in BSCB after lactate administration visualized by TEM. Scale bar=2.0 μm. *n*=6 per group. **E** Protein levels of ZO-1, Occludin, Claudin-5, Glut1, Eno1 and LDHα detected by Western blot (left) and their quantitative analyses (right). Samples of SR + PBS and SR + NaLA groups were collected on Day 1 and those of SI + PBS and SI + NaLA groups were collected on Day 7. *n*=3 per group. *p<0.05, **p<0.01, ***p<0.001, ns: no significance

**Fig. 6:**
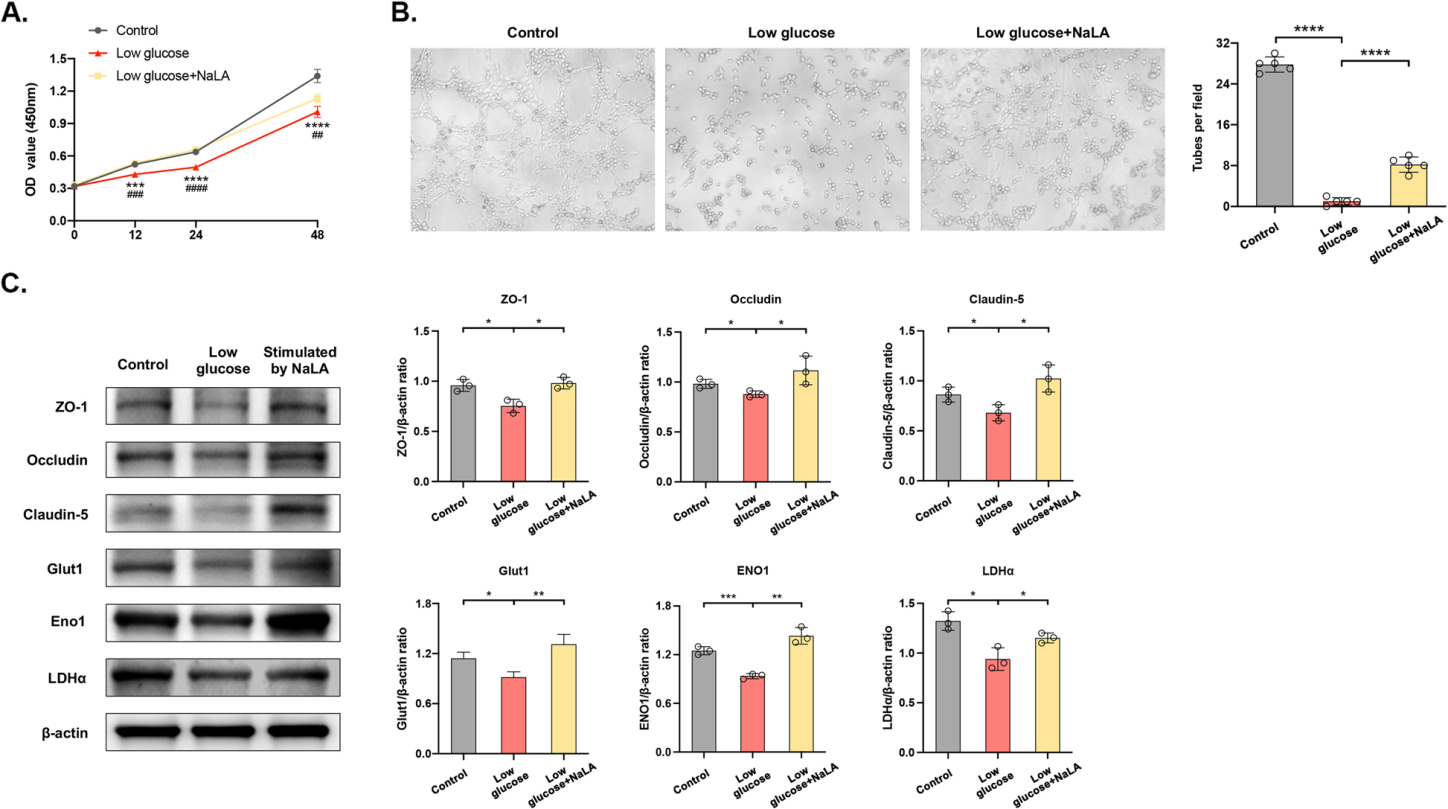
Lactate administration rescues functions and glycolysis of HUVECs. **A** Proliferative rate of HUVECs at different time points detected by CCK-8 assay. *n*=5 per group. Control *vs.* Low glucose: ***p<0.001, ****p<0.0001; Low glucose *vs.* Low glucose + NaLA: ^###^*p*<0.001, ^####^*p*<0.0001. **B** Tube formation by HUVECs in five random fields. *n*=5 per group. ****p<0.0001. **C** Protein levels of ZO-1, Occludin, Claudin-5, Glut1, Eno1 and LDHα detected by Western blot (left) and their quantitative analyses (right). *n*=3 per group. *p<0.05, **p<0.01, ***p<0.001

Glycolysis disturbance in ECs resulted in the deficiency of lactic acid for pericytes to support the barrier, and impairment of the proliferation and functions of ECs [17]. The supplementation of sodium lactate is theoretically able to repair SR-induced damages to the barriers and hyperalgesia. Compared with those administrated with PBS, SR mice administrated with sodium lactate presented a higher level of lactic acid, suggesting the spread of sodium lactate into the spinal cord [*p* < 0.05] (Fig. 5A). During the process, the recovery time of hyperalgesia in mice of both SR and SR + I groups was significantly shortened [SR + PBS group vs. SR + NaLA group: *p* < 0.05 on Day 1, 2, 5, 7, *p* < 0.01 on Day 3; SI + PBS group vs. SI + NaLA group: *p* < 0.05 on Day3, *p* < 0.01 on Day 7, *p* < 0.001 on Day 5, 9] (Fig. 5B). After the treatment of sodium lactate, *in vivo* imaging examinations revealed a better structure of BSCB and BBB in SR mice compared with those of the SR + PBS group [*p* < 0.05] (Fig. 5C). TEM consistently visualized the recovery of the tight junctions of the BSCB with a clear and complete structure (Fig. 5D). The protein expression levels of tight junctions were significantly upregulated by the treatment of sodium lactate, whereas the levels of inflammatory factors were significantly reduced [SR + PBS group vs. SR + NaLA group: ZO-1: *p* < 0.01, Occludin: *p* < 0.05, Claudin-5: *p* < 0.001, p-NR2B: *p* < 0.05, IL-1β: *p* < 0.05, IL-6: *p* < 0.01, TNF-α: *p* < 0.01; SI + PBS group vs. SI + NaLA group: ZO-1: *p* > 0.05, Occludin: *p* < 0.01, Claudin-5: *p* < 0.05, p-NR2B: *p* < 0.05, IL-1β: *p* < 0.001, IL-6: *p* < 0.01, TNF-α: *p* < 0.001] (Fig. 5E). Meanwhile, we also injected normal mice with sodium lactate of the same regimen and found that compared with the non-injection group, there was no significant difference in the levels of tight junction proteins and glycolysis related proteins between the two groups [ZO-1, Occludin, Claudin-5, Glut1, ENO1, LDHα: *p* > 0.05] (Additional file 4: Fig. S4A). In the *in vitro* HUVECs induced with 2 μM sodium lactate in DMEM low glucose solution for 2 days, the impaired proliferative and angiogenic abilities of HUVECs were remarkably recovered [CCK8 assay: Control group vs. Low glucose group: *p* < 0.001 on 12h, *p* < 0.0001 on 24h and 48h; Low glucose group vs. Low glucose + NaLA group: *p* < 0.001 on 12h, *p* < 0.0001 on 24h, *p* < 0.01 on 48h; complete tubular structures formed: Control group vs. Low glucose group*: p* < 0.0001; Low glucose group vs. Low glucose + NaLA group: *p* < 0.0001] (Fig. 6A-B), and the expression levels of tight junctions and glycolytic enzymes basically returned to normal [Control group vs. Low glucose group: ZO-1: *p* < 0.05, Occludin: *p* < 0.05, Claudin-5: *p* < 0.05, Glut1: *p* < 0.05, ENO1: *p* < 0.001, LDHα: *p* < 0.05; Low glucose group vs. Low glucose + NaLA group: ZO-1: *p* < 0.05, Occludin: *p* < 0.05, Claudin-5: *p* < 0.05, Glut1: *p* < 0.01, ENO1: *p* < 0.01, LDHα: *p* < 0.05] (Fig. 6C). Similarly, HUVECs did not have significant difference of proliferative and angiogenic abilities with that induced with sodium lactate in DMEM normal medium [complete tubular structures formed: *p* > 0.05; CCK8 assay:* p* > 0.05 on 12, 24 and 48h] (Additional file 4: Fig. S4B, C). We have validated that the supplementation of sodium lactate relieved SR-induced hyperalgesia and EC dysfunction, which further favored the repair of the BSCB. More importantly, glycolysis disorders were a vital event for the destruction of BSCB.

### The mTOR signaling pathway participates in the damage to the BSCB following EC dysfunction

**Fig. 7:**
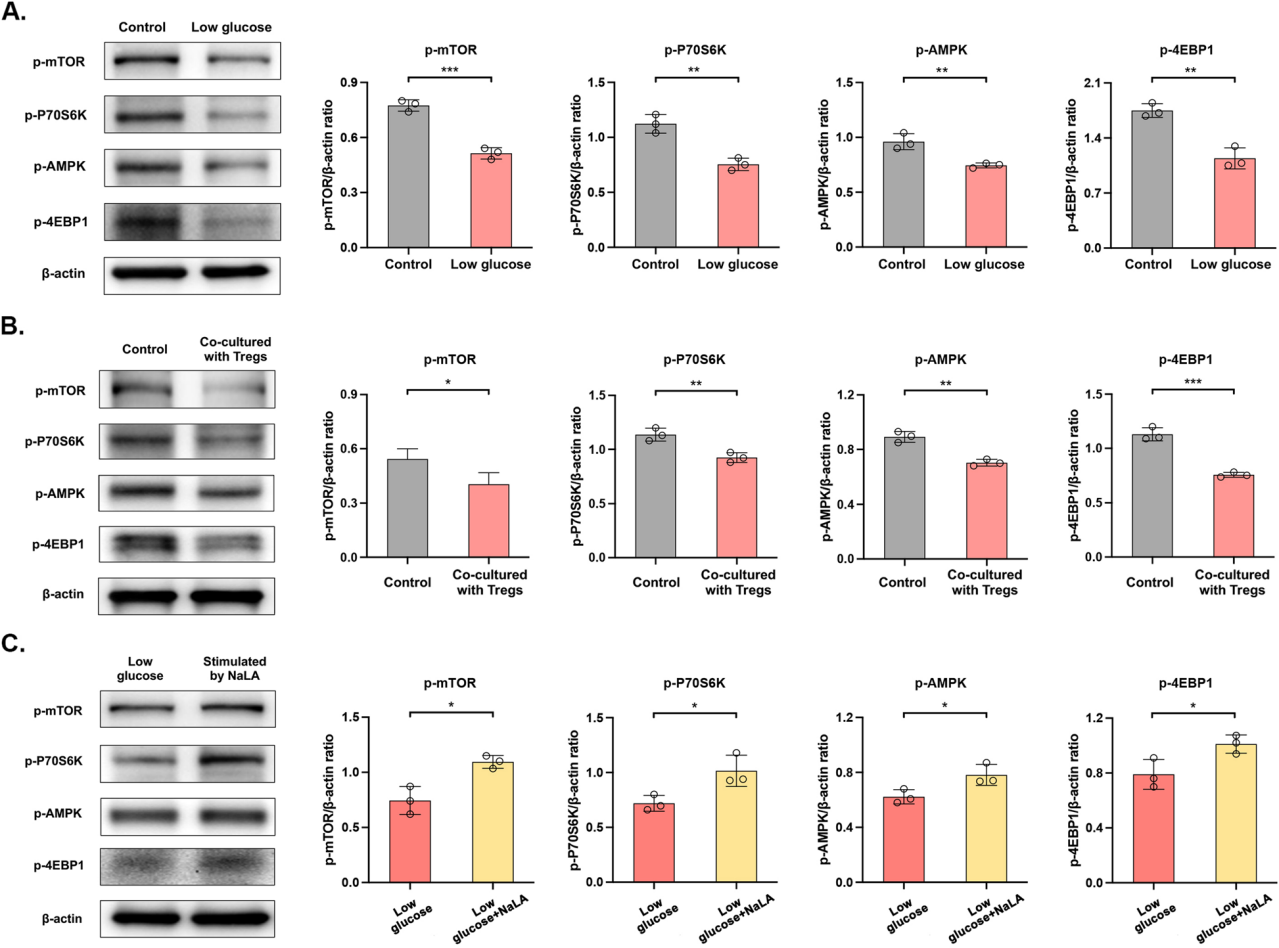
The mTOR signaling pathway participates in the damage to the BSCB damage following EC dysfunction. **A**, **B**, **C** Protein levels of p-mTOR, p-P70S6K, p-AMPK, p-4EBP1 detected by Western blot (left) and their quantitative analyses (right). *p<0.05, **p<0.01, ***p<0.001

**Fig. 8:**
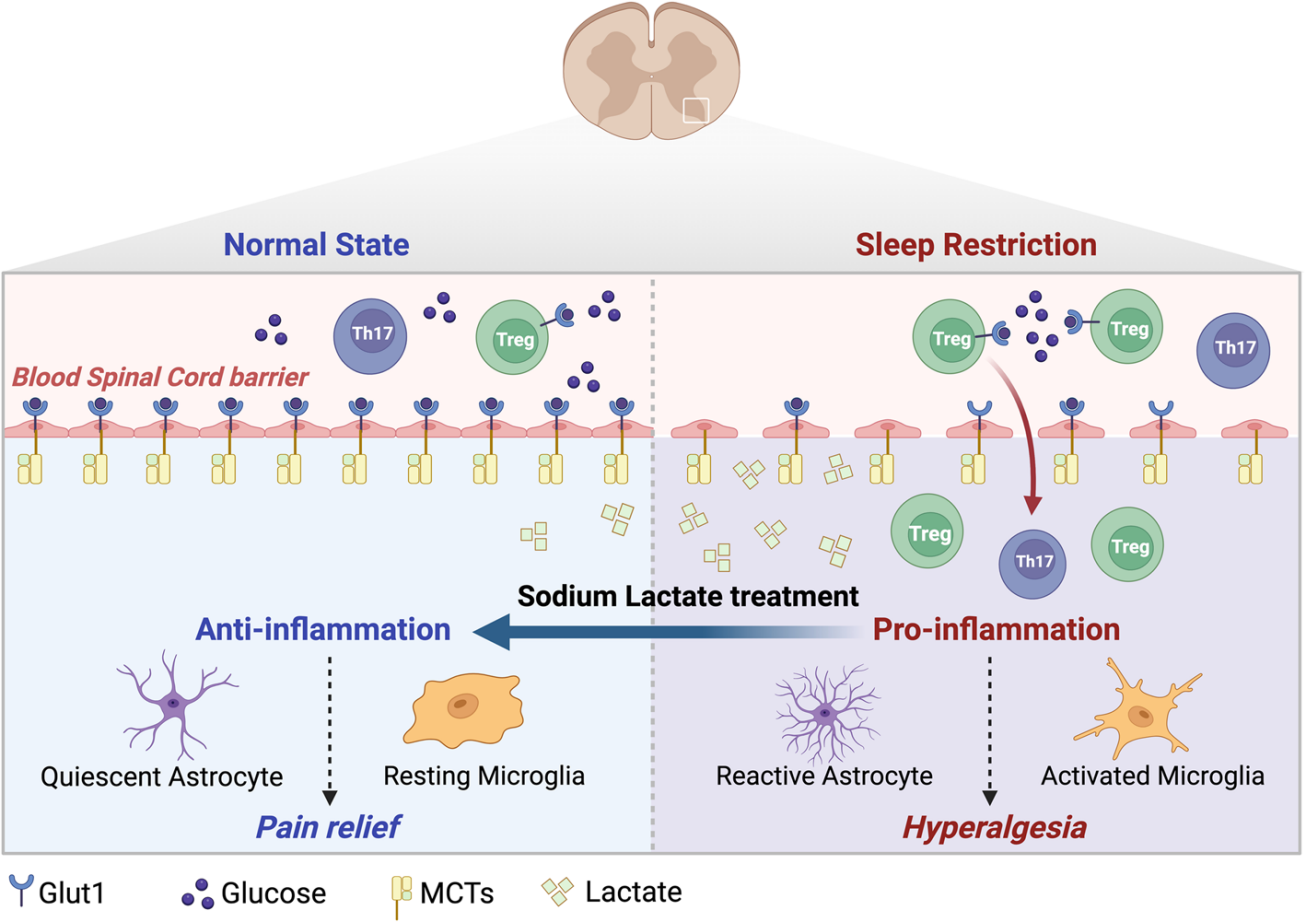
Schematic diagram of the current study. Affected by glycolysis disorders of ECs due to glucose competition with infiltrating Tregs through regulating the mTOR signaling pathway, hyperalgesia induced by 48-h SR is attributed to neuroinflammation and damages to the barriers, which can be relieved by lactate supplementation

The mTOR signaling pathway is important in the metabolism of ECs. Expression levels of key proteins in the mTOR signaling pathway were significantly downregulated in ECs with glucose deficiency or treated with 2-DG than those of controls [Control group vs. Low glucose group: p-mTOR: *p* < 0.001, p-P70S6K: *p* < 0.01, p-AMPK: *p* < 0.01, p-4EBP1: *p* < 0.01; Control group vs. Stimulated by 2-DG group: p-mTOR: *p* < 0.05, p-P70S6K: *p* < 0.05, p-AMPK: *p* < 0.05, p-4EBP1: *p* < 0.001] (Fig. 7A and S2E). Similar results were obtained in HUVECs co-cultured with Tregs [p-mTOR: *p* < 0.05, p-P70S6K: *p* < 0.01, p-AMPK: *p* < 0.01, p-4EBP1: *p* < 0.001] (Fig. 7B). Later, expression levels of key proteins in the mTOR signaling pathway of HUVECs treated with sodium lactate returned to the similar levels as those induced in the normal medium [p-mTOR: *p* < 0.05, p-P70S6K: *p* < 0.05, p-AMPK: *p* < 0.05, p-4EBP1: *p* < 0.05] (Fig. 7C). Hence, the mTOR signaling pathway was confirmed to participate in EC dysfunction caused by infiltrating Tregs following glycolytic disturbances (Fig. 8).

## Discussion

Sleep loss may cause the accumulation of proinflammatory cytokines in blood and this may be cause by Enhanced efflux through the BBB [40]. Our previous study found that SR-induced hyperalgesia is associated with the inflammation of the spinal dorsal cord [27]. Consistently, our findings of increased levels of pro-inflammatory factors in the spinal cord and activation of glia confirmed the previous conclusion. Immune cells play a critical role in promoting neuroinflammation and development of pain [41–43]. The number of T cells was found elevated in mouse spinal cord after 48-h SR, especially the infiltrating Tregs. Controversially, Tregs can either inhibit the inflammation [44], or stimulate the progression of bone cancer pain (unpublished data in our research group). Nathan et al. [45] reported different changes in meningeal and lymph node immune cell profiles between male and female mice. Inflammation is a process influenced by metabolism [46, 47], and we therefore speculated that the different functions of Tregs may be attributed to the metabolic mode. Tregs used to be considered to regulate energy metabolism through oxidative phosphorylation of glucose and fatty acids. The glucose metabolism pathway of Tregs is later identified to experience a temporary environment-related metabolic transformation into glycolysis, that is, metabolic reprogramming, in specific proliferative environments (e.g., inflammation and tumours) [48, 49]. Peripheral Tregs in tumor patients expressing high levels of Glut1 have higher glucose uptake and glycolysis flux than those of effector T cells [50, 51]. Our data showed the increased level of glycolysis in the infiltrating Tregs, downregulated Glut1 in ECs and dysfunction of ECs, suggesting that SR-induced neuroinflammation was linked with the competition between Tregs and ECs for glucose (glycolysis).

Glycolysis is an essential process for ECs, and the lactate produced by them can be used by the surrounding cells. Relying on the locally produced lactate, neurons and macrophages maintain their energy requirements. Zhang et al. [52] found that endothelium-generated lactate induces macrophages to promote muscle regeneration during ischemia. Lee et al. [18] found that the lack of endothelium-derived lactate leads to pericyte loss from the vessel wall, thereby increasing the BBB permeability. High-dose oral lactate supplementation significantly improves the BBB integrity in Glut1^iECKO^ mice although it does not enhance the mortality. In the present study, the function of ECs and the integrity of the BSCB were significantly improved after the treatment of sodium lactate in SR mice. More importantly, it significantly shortened SR-induced prolongation of postoperative pain. Lactate supplementation alone may not be sufficient to prevent severe cases, it is expected beneficial for postoperative patients with SR.

Illustrating the mechanism of glycolysis in Tregs and ECs of mice with SR-induced hyperalgesia favors the identification of therapeutic targets. The mTOR signaling pathway is a key negative regulator for Tregs [53–55]. Stimuli in T cells activates the PI3K/AKT signaling pathway. The AKT kinase further stimulates the formation of a basic nutrition-sensitive complex by mTORC1, which triggers glucose transport and aerobic glycolysis through upregulating Glut1 and accelerating its transportation on cell membrane [56]. In contrast, stimuli in T cells impair the function of ECs. In mouse brain microvascular endothelial cells (BMVECs) exposed to oxygen-glucose deprivation (OGD), the protein levels of tight junction markers are significantly downregulated but rescued by the treatment of AKT and mTOR inhibitors [57]. Moreover, the activated PI3K-AKT-mTOR pathway exerts a protective role in the endothelial barrier integrity [58]. Our findings showed the inhibition of the mTOR signaling pathway and decreases in tight junctions in ECs induced with DMEM low glucose solution or co-cultured with Tregs, which were effectively reversed by the treatment of sodium lactate. Taken together, our study highlighted the effect of Tregs on glycolysis and barrier integrity of ECs through regulating the mTOR signaling pathway.

The study has some limitations. In the current investigation, we used the 48 h SR model as an acute sleep loss model. Acute sleep loss means that individuals remain awake for a short period of time (usually 24 - 72 hours) [30, 40, 59]. As animals have a homeostatic mechanism that drives robust recovery after an initial loss of sleep [59, 60], we found that 48 h SR could significantly suppress all phases of sleep and cause hyperalgesia. What’ more, the 48-h SR could not cause mice die with clear environment and this is consistent with Sang’s work [40]. Although the mice were not completely sleep abolished, they were able to stay in sleep loss for a period. Of course, a more efficient experimental paradigm for continuous SD is necessary to explored [40]. The *in vivo* regulatory effect of the mTOR signaling pathway and Tregs depletion in SR mice, however, was not performed due to the challenges in isolating epithelial cells of the spinal cord and cultivating tool mice in a short period. It is important to do further investigation about this question in our future work.

## Conclusions

Hyperalgesia induced by 48-h SR is attributed to neuroinflammation and damages to the barriers, which can be relieved by lactate supplementation (Fig. 8). The hyperalgesia is affected by glycolysis disorders of ECs due to glucose competition with infiltrating Tregs around the barrier via regulating the mTOR signaling pathway.

### Supplementary Information

**Additional file 1: Fig. S1.** 48-h SR causes and prolongs neuroinflammation. (A) Protein levels of p-NR2B, IL-1β, IL-6 and TNF-αdetected by Western blot (left) and their quantitative analyses (right). Samples in both groups were collected on Day 7 post-incision. *n*=3 per group. *p<0.05, **p<0.01. B The populations of astrocytes and microglia in mouse spinal cord. Representative flow cytometric analysis of astrocytes (GFAP^+^ cells) and microglia (CD45^int^CD11b^hi^ cells) (left) and their proportions on Day 1 (right). *n*=3 per group. *p<0.05.

**Additional file 2:** **Fig. S2.** 2-DG treatment inhibits glycolysis in HUVECs. (A) Protein levels of ZO-1, Occludin, Claudin-5, Glut1, Eno1 and LDHα detected by Western blot (left) and their quantitative analyses (right). *n*=3 per group. *p<0.05, **p<0.01, *** *p*<0.001. (B) Tube formation by HUVECs in five random fields. *n*=5 per group. ****p<0.0001. (C) Proliferative rate of HUVECs at different time points detected by CCK-8 assay. *n*=5 per group. ***p<0.001. (D) The standard curve of glycolysis levels in HUVECs (left), optical density (middle) and the level of L-Lactate by conversion (right). *n*=3 per group. ****p<0.0001. (E) Protein levels of p-mTOR, p-P70S6K, p-AMPK and p-4EBP1 detected by Western blot (left) and their quantitative analyses (right). *n*=3 per group. *p<0.05, ***p<0.001.

**Additional file 3:** **Fig. S3.** Infiltrating T cell subsets in spinal cord of SR mice. (A) Representative flow cytometric analysis of CD3^+^ T cells and CD4^+^IL17^+^ T cells (Th17 cells) (left) and their proportions on Day 1 post-SR (right). *n*=8 per group. ****p<0.0001, ns: no significance. (B) The flow cytometric analysis of CD4^+^CD25^+^ T cells sorted from mouse spleens by Dynabeads™ FlowComp™ Mouse CD4^+^CD25^+^ Treg Cells Kit. (C) Expression level of Foxp3 in CD4^+^CD25^+^ T cells sorted from mouse spleens detected by flow cytometry. (D) Schematic diagram of the co-culture system. (E) Expression level of Foxp3 in Tregs before and after co-culturing detected by flow cytometry.

**Additional file 4:** **Fig. S4.** NaLA administration makes no significance on normal mice and HUVECs. (A) Protein levels of ZO-1, Occludin, Claudin-5, Glut1, Eno1 and LDHα detected by Western blot (left) and their quantitative analyses (right). *n*=3 per group. ns, *p*>0.05. (B) Tube formation by HUVECs in five random fields. *n*=5 per group. ns, *p*>0.05. (C) Proliferative rate of HUVECs at different time points detected by CCK-8 assay. *n*=5 per group. ns, *p*>0.05.

**Additional file 5.**

**Additional file 6.**

## Data Availability

All data generated or analyzed during this study are included in this published article.

## Abbreviations

SR: Sleep restriction
TEM: Transmission electron microscopy
BSCB: Blood-spinal cord barrier
BBB: Blood-brain barrier
PWMT: Paw withdrawal mechanical threshold
Tregs: Regulatory T cells
ECs: Endothelial cells
CNS: Central nervous system
Glut1: Glucose transporter 1
ENO1: Enolase 1
LDHα: Lactate dehydrogenase α
mTOR: Mammalian target of rapamycin
SPF: Specific pathogen-free
RT: Room temperature
