# Supplementary figures and images for: Glucose competition between endothelial cells in the blood-spinal cord barrier and infiltrating regulatory T cells is linked to sleep restriction-induced hyperalgesia
Source: BMC Med. 2024 May 7;22:189. doi: 10.1186/s12916-024-03413-z (PMC11077863; doi:10.1186/s12916-024-03413-z)

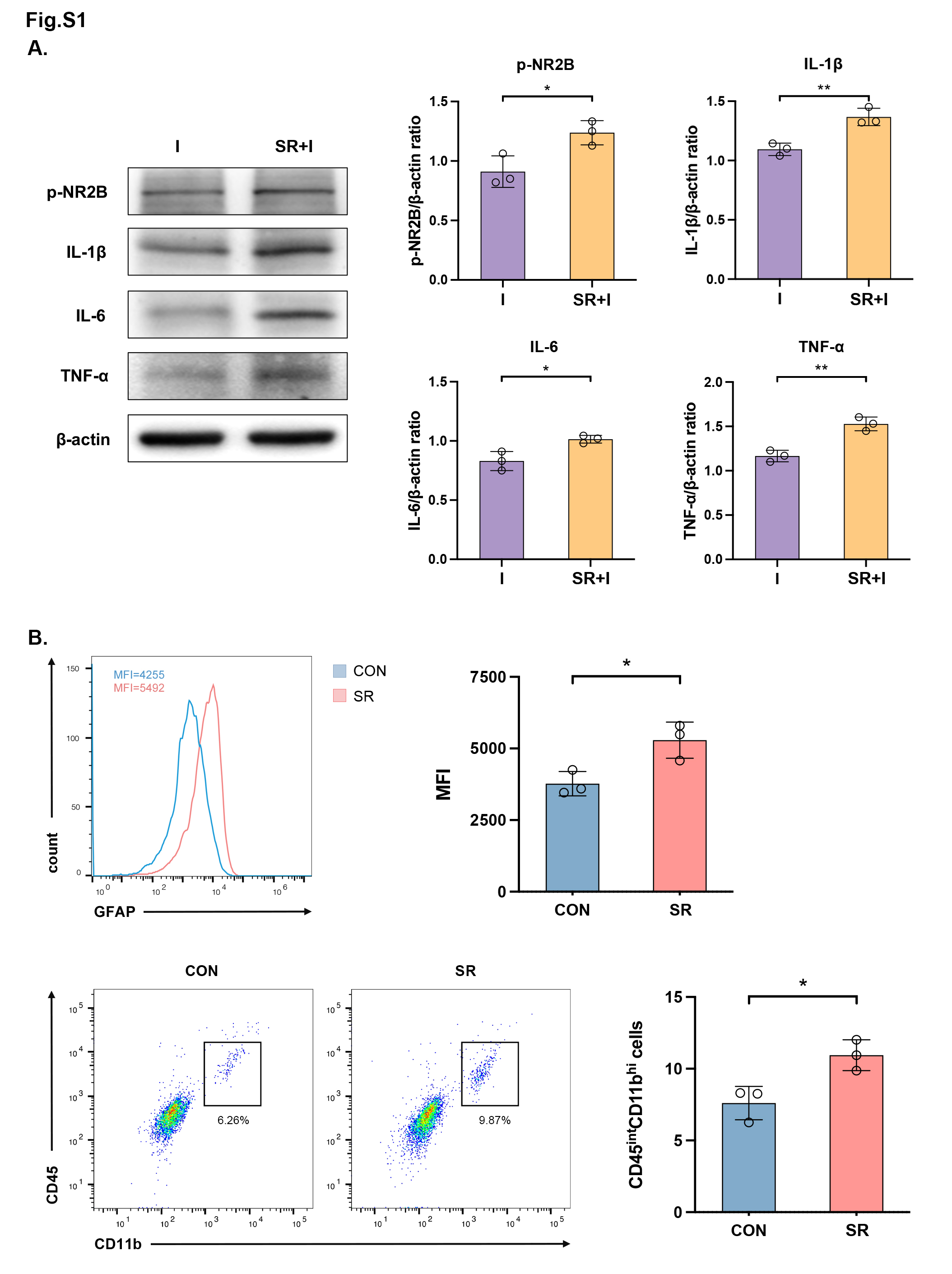

Supplement: Supplementary file 1 — Additional file 1: Fig. S1. 48-h SR causes and prolongs neuroinflammation. (A) Protein levels of p-NR2B, IL-1β, IL-6 and TNF-αdetected by Western blot (left) and their quantitative analyses (right). Samples in both groups were collected on Day 7 post-incision. n=3 per group. *p<0.05, **p<0.01. B The populations of astrocytes and microglia in mouse spinal cord. Representative flow cytometric analysis of astrocytes (GFAP+ cells) and microglia (CD45intCD11bhi cells) (left) and their proportions on Day 1 (right). n=3 per group. *p<0.05. [file 12916_2024_3413_MOESM1_ESM.jpg]

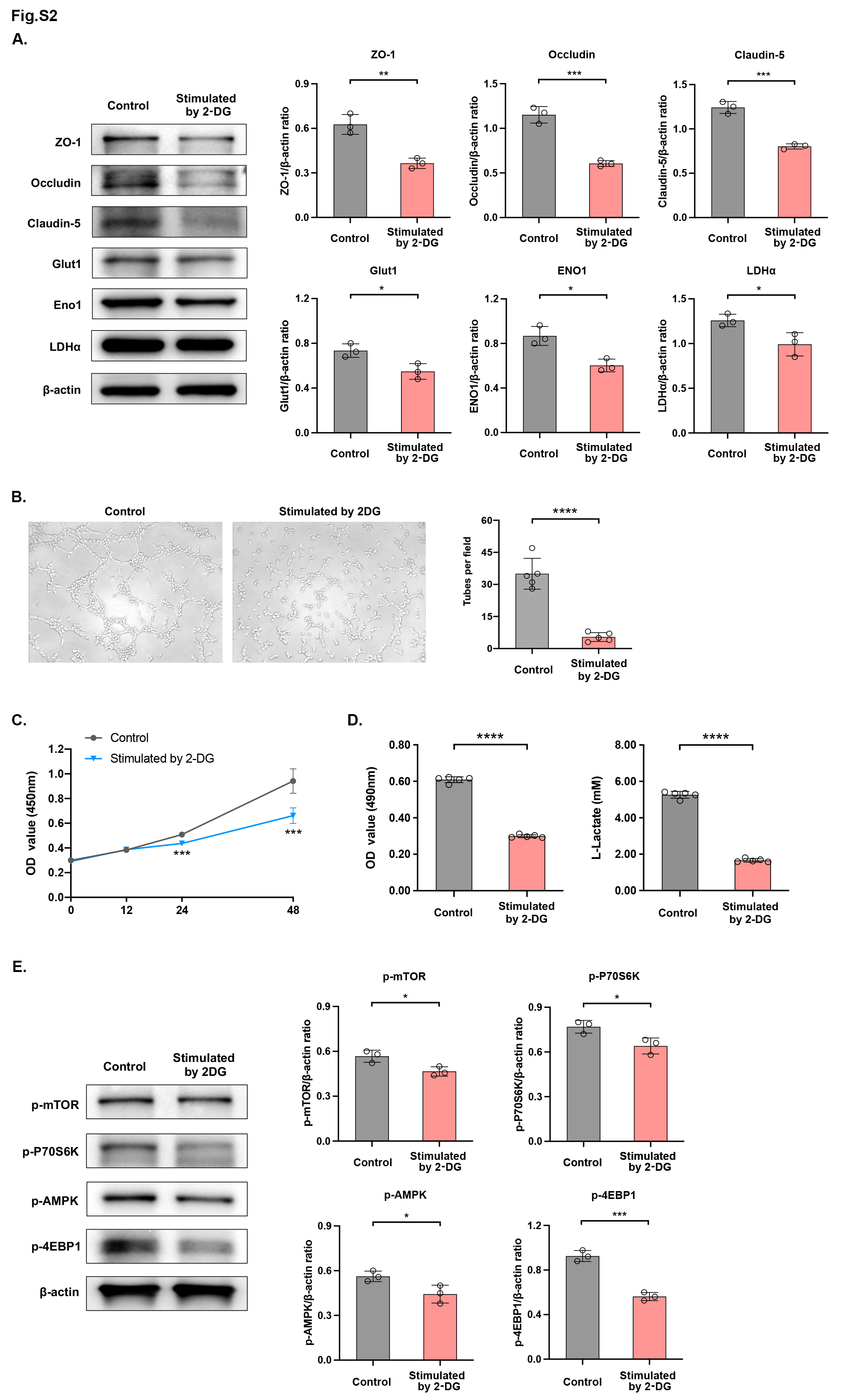

Supplement: Supplementary file 2 — Additional file 2: Fig. S2. 2-DG treatment inhibits glycolysis in HUVECs. (A) Protein levels of ZO-1, Occludin, Claudin-5, Glut1, Eno1 and LDHα detected by Western blot (left) and their quantitative analyses (right). n=3 per group. *p<0.05, **p<0.01, *** p<0.001. (B) Tube formation by HUVECs in five random fields. n=5 per group. ****p<0.0001. (C) Proliferative rate of HUVECs at different time points detected by CCK-8 assay. n=5 per group. ***p<0.001. (D) The standard curve of glycolysis levels in HUVECs (left), optical density (middle) and the level of L-Lactate by conversion (right). n=3 per group. ****p<0.0001. (E) Protein levels of p-mTOR, p-P70S6K, p-AMPK and p-4EBP1 detected by Western blot (left) and their quantitative analyses (right). n=3 per group. *p<0.05, ***p<0.001. [file 12916_2024_3413_MOESM2_ESM.jpg]

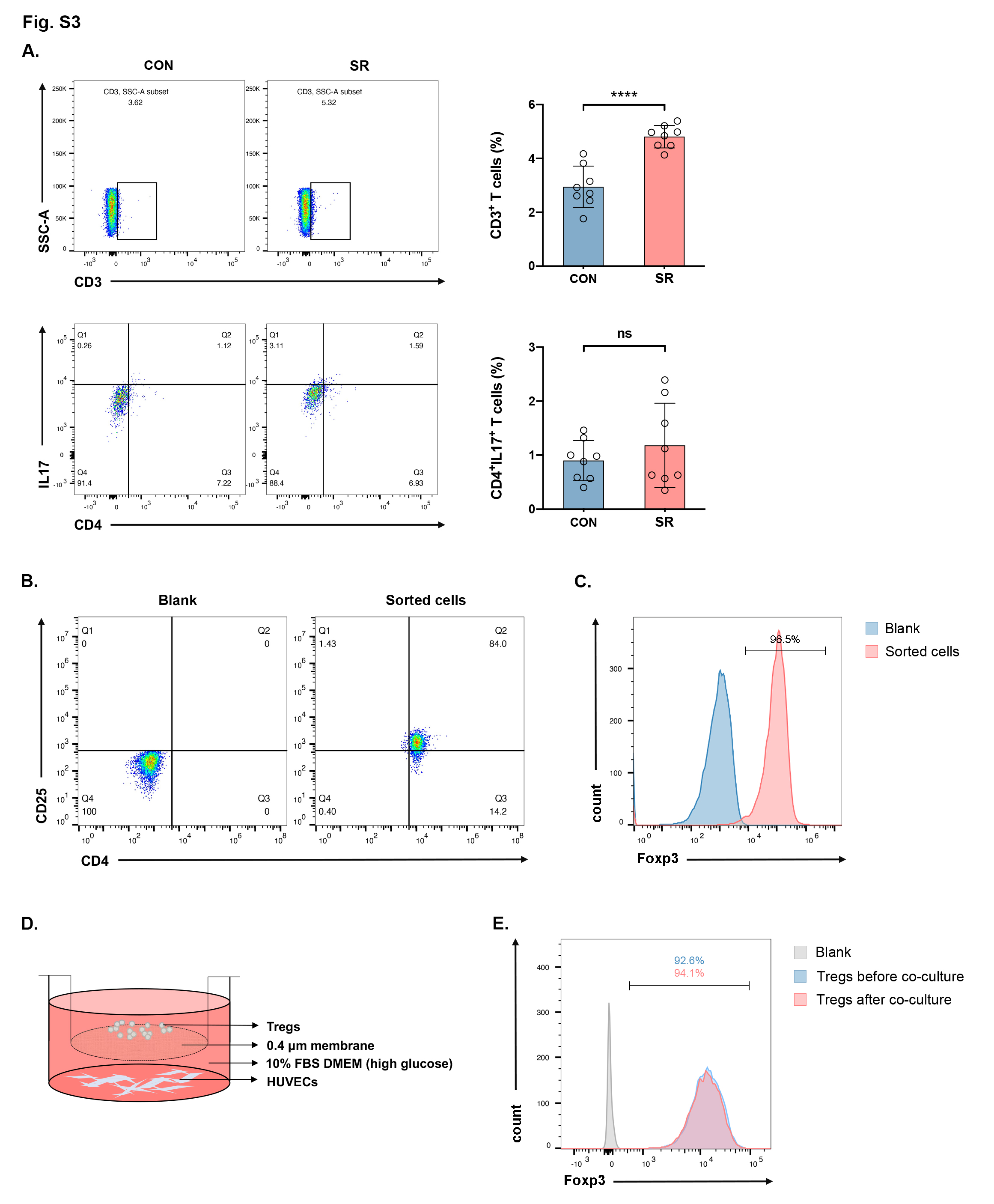

Supplement: Supplementary file 3 — Additional file 3: Fig. S3. Infiltrating T cell subsets in spinal cord of SR mice. (A) Representative flow cytometric analysis of CD3+ T cells and CD4+IL17+ T cells (Th17 cells) (left) and their proportions on Day 1 post-SR (right). n=8 per group. ****p<0.0001, ns: no significance. (B) The flow cytometric analysis of CD4+CD25+ T cells sorted from mouse spleens by Dynabeads™ FlowComp™ Mouse CD4+CD25+ Treg Cells Kit. (C) Expression level of Foxp3 in CD4+CD25+ T cells sorted from mouse spleens detected by flow cytometry. (D) Schematic diagram of the co-culture system. (E) Expression level of Foxp3 in Tregs before and after co-culturing detected by flow cytometry. [file 12916_2024_3413_MOESM3_ESM.jpg]

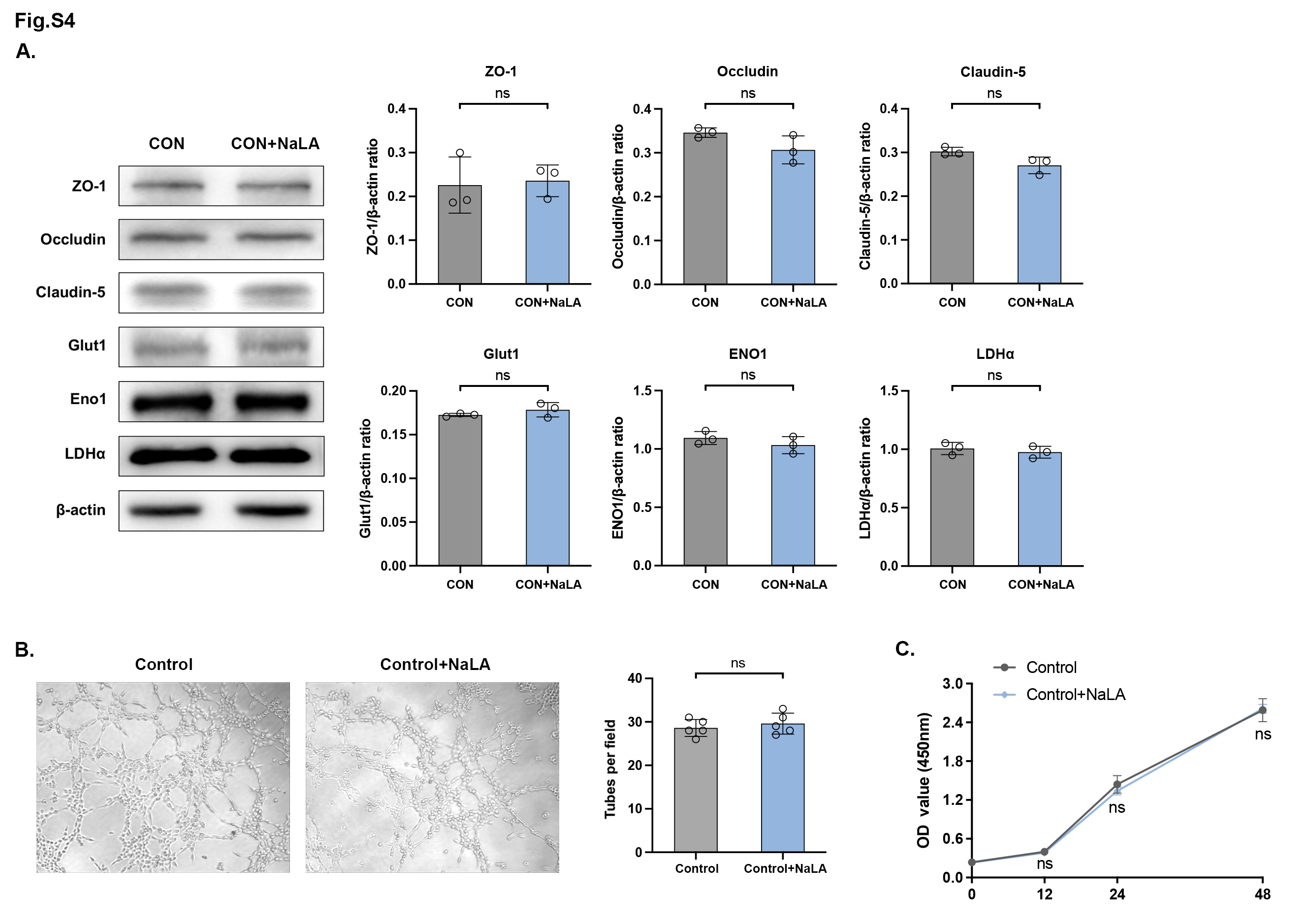

Supplement: Supplementary file 4 — Additional file 4: Fig. S4. NaLA administration makes no significance on normal mice and HUVECs. (A) Protein levels of ZO-1, Occludin, Claudin-5, Glut1, Eno1 and LDHα detected by Western blot (left) and their quantitative analyses (right). n=3 per group. ns, p>0.05. (B) Tube formation by HUVECs in five random fields. n=5 per group. ns, p>0.05. (C) Proliferative rate of HUVECs at different time points detected by CCK-8 assay. n=5 per group. ns, p>0.05. [file 12916_2024_3413_MOESM4_ESM.jpg]

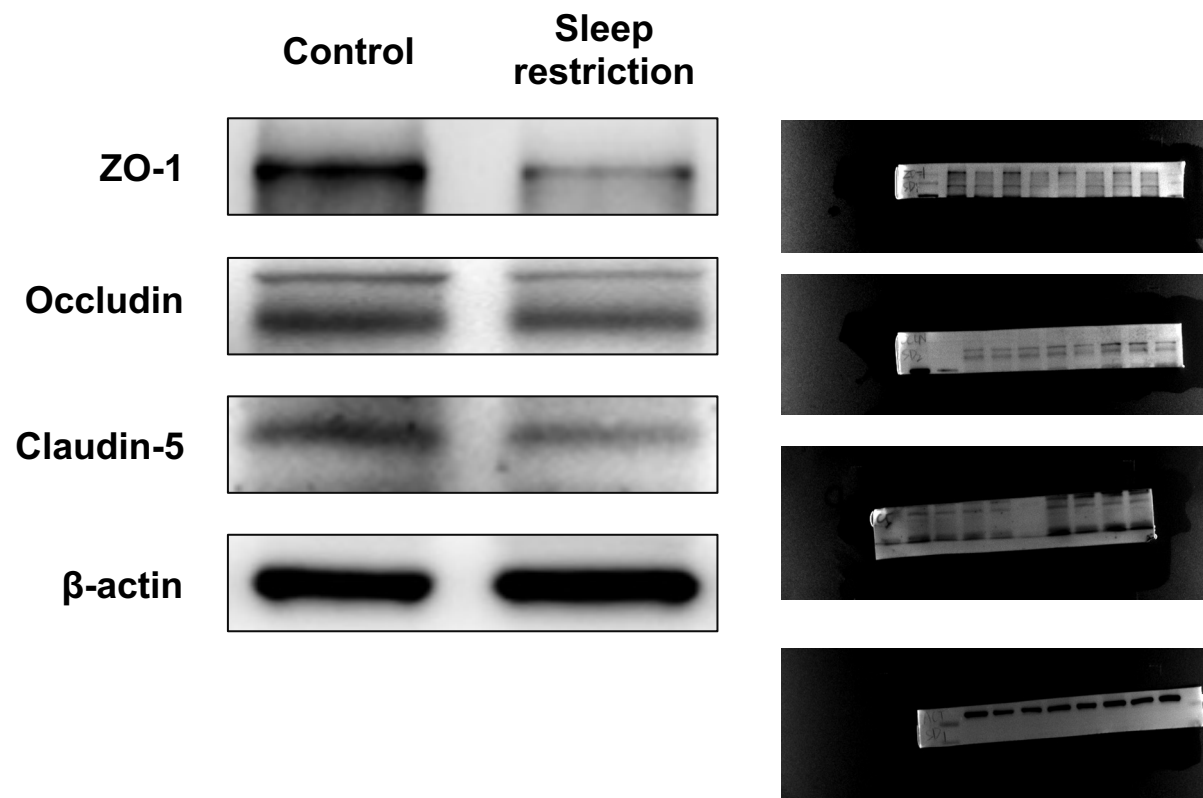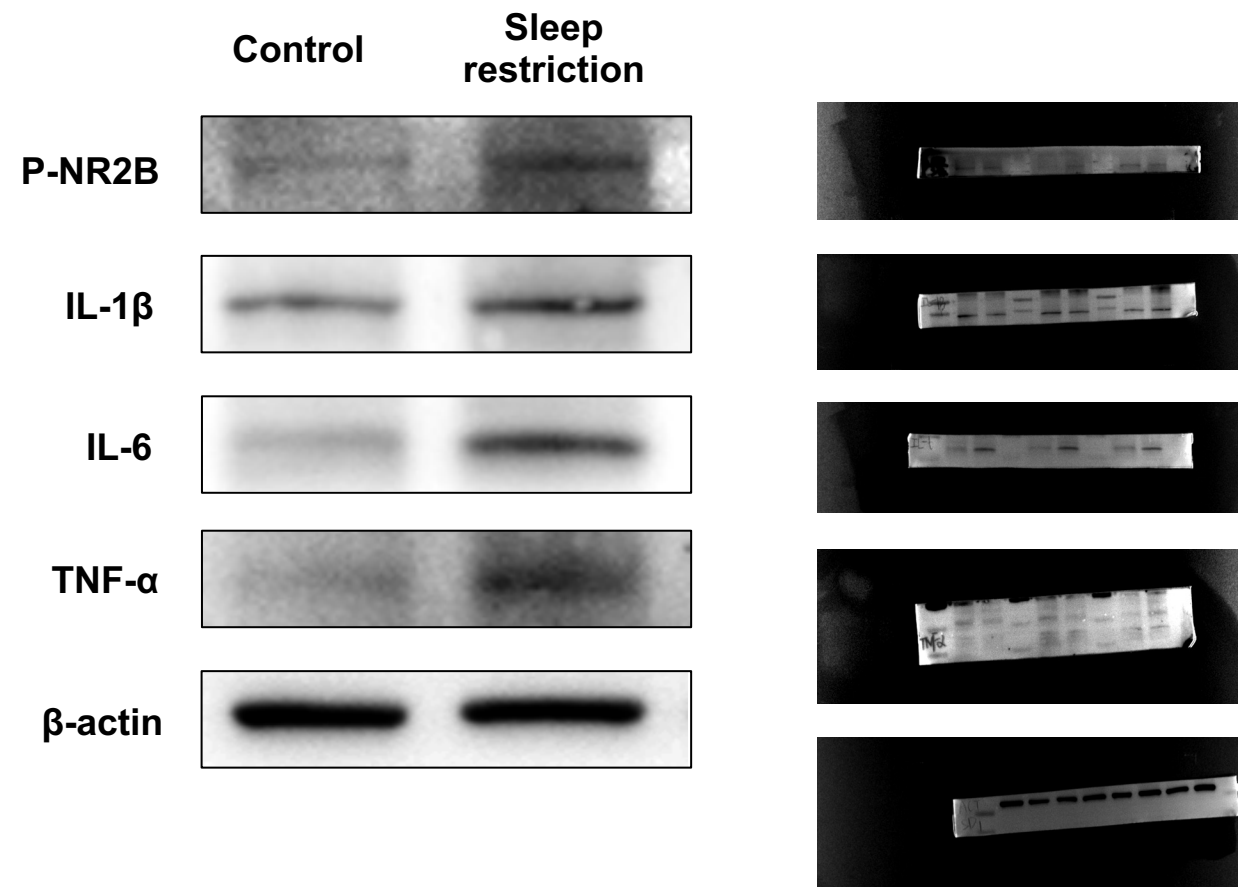

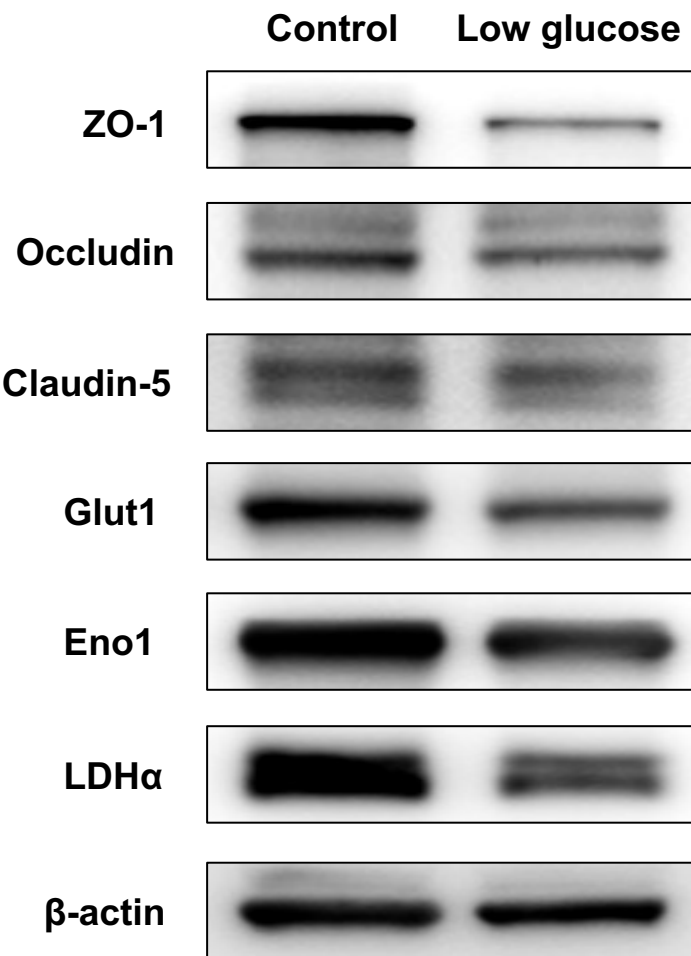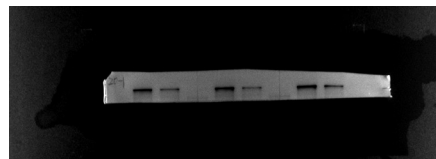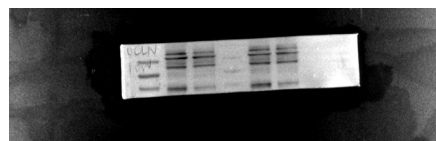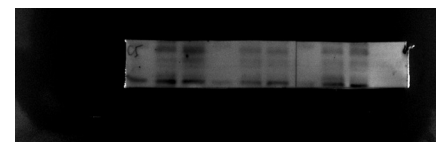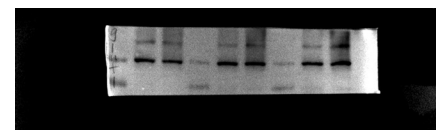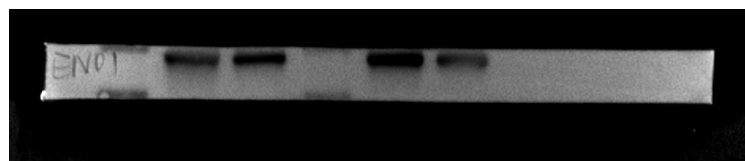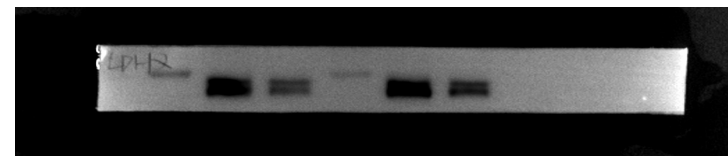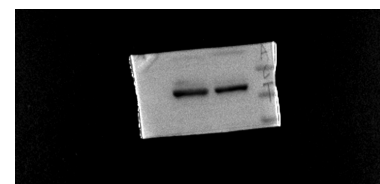

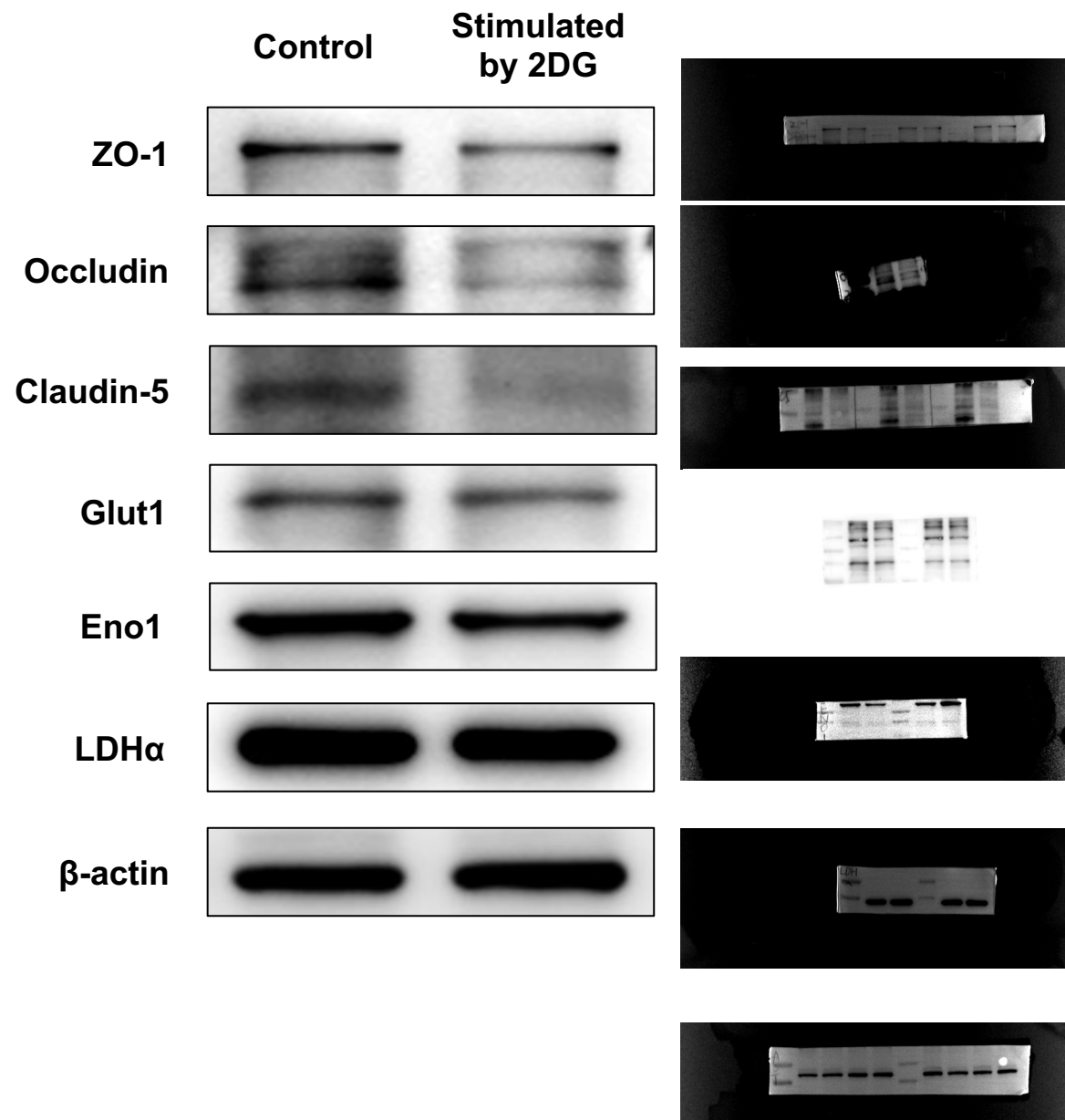

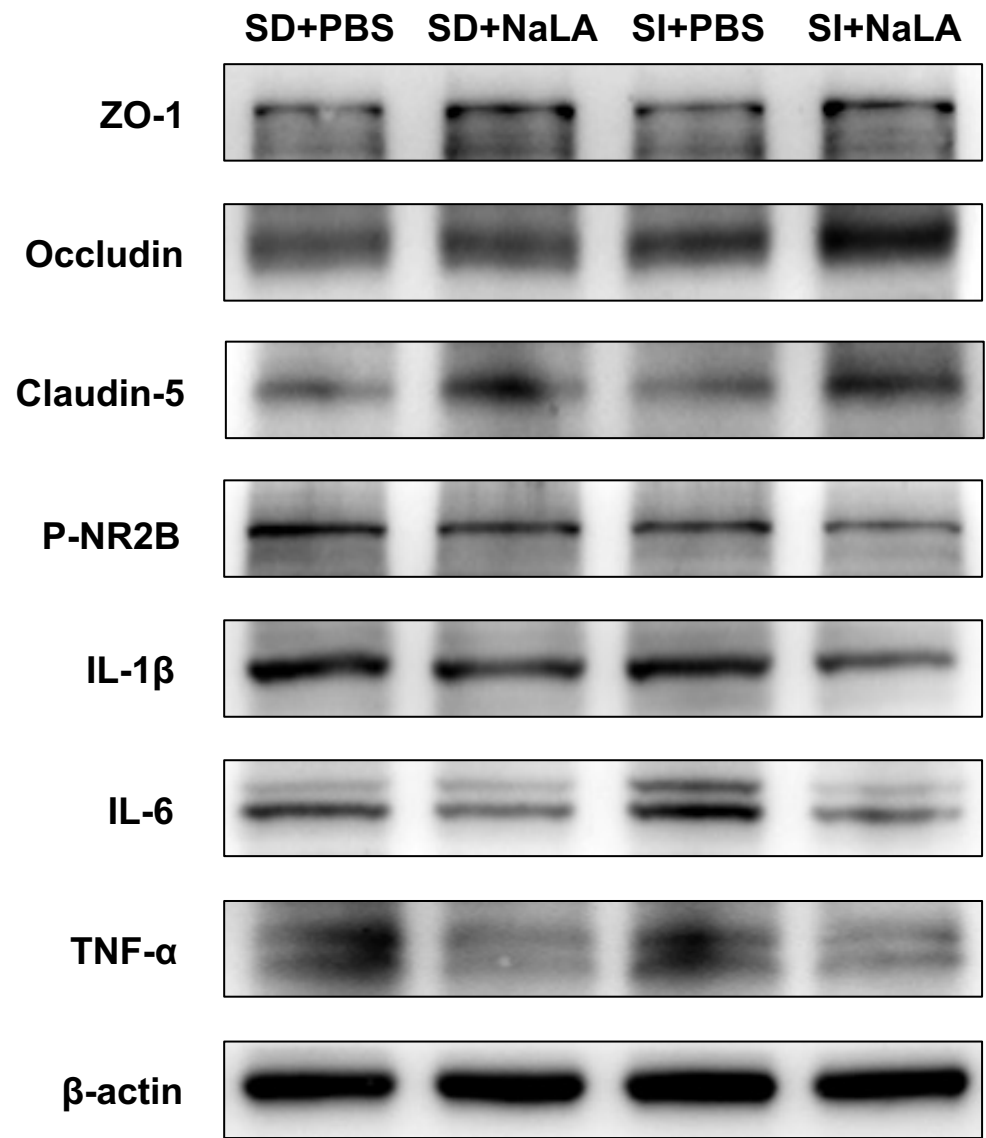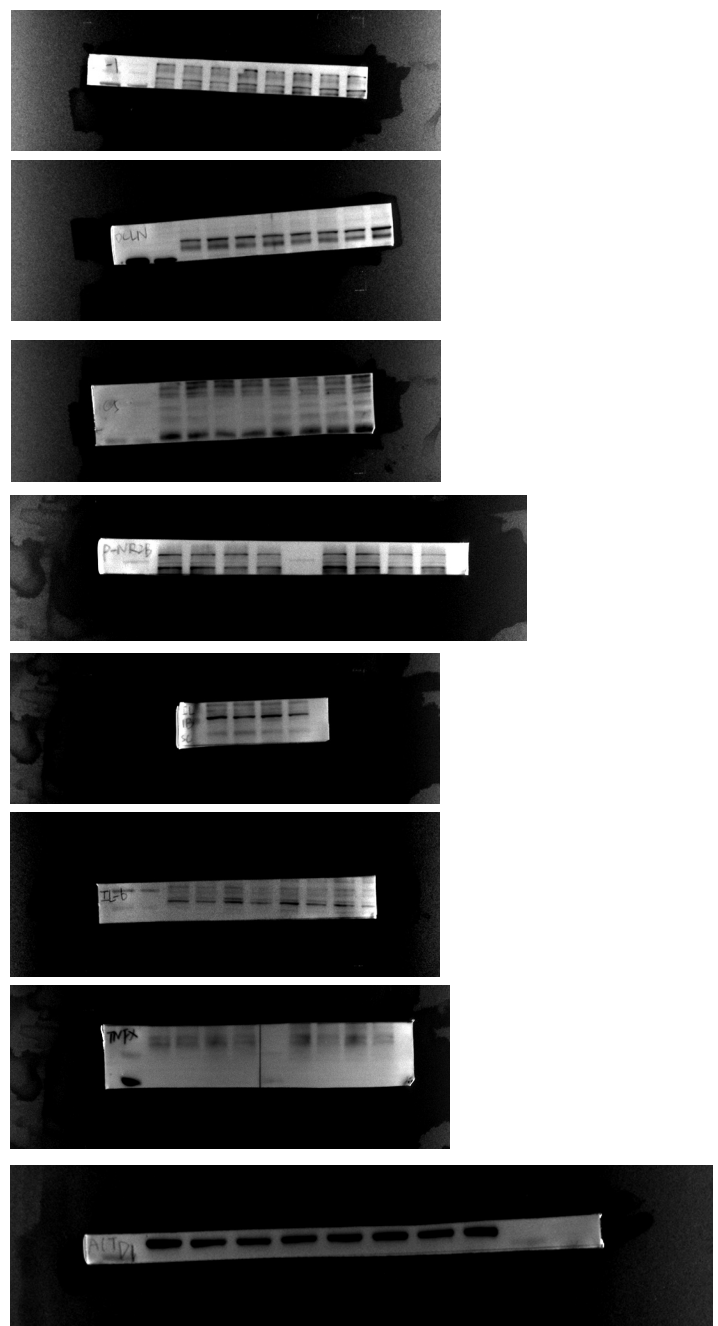

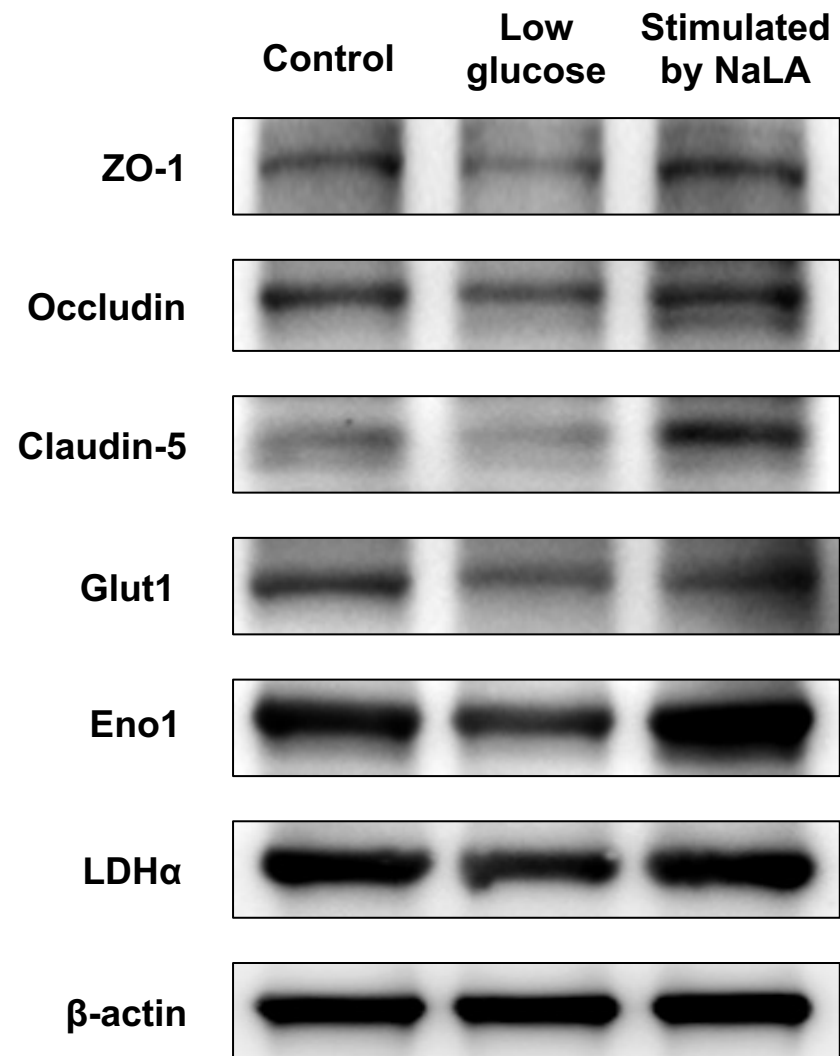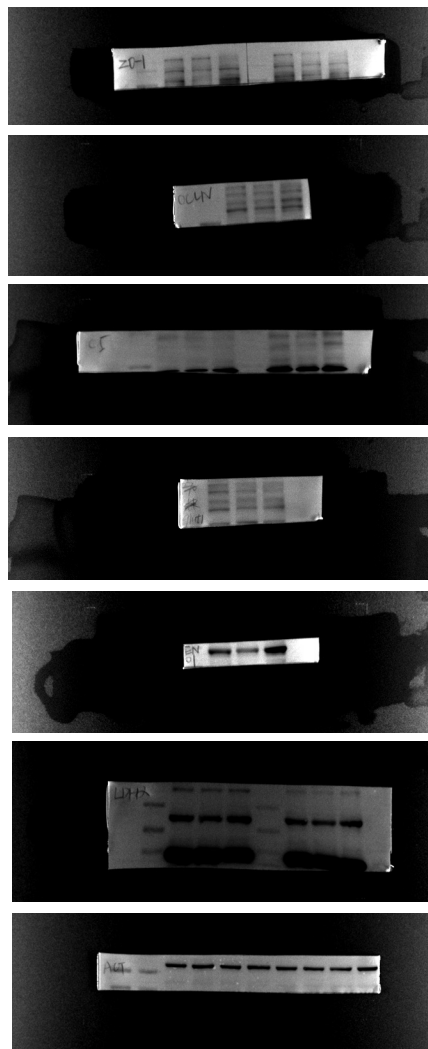

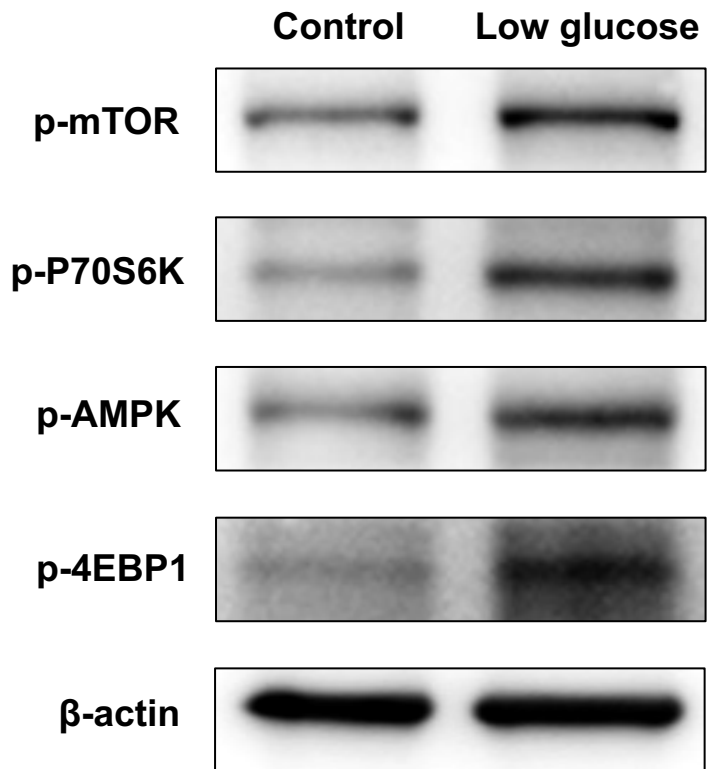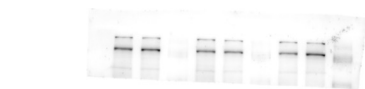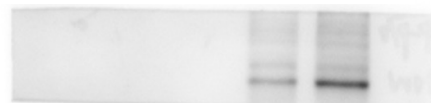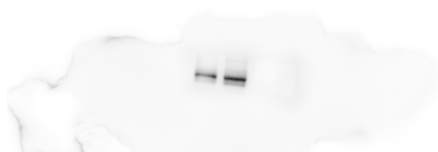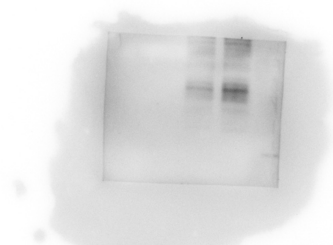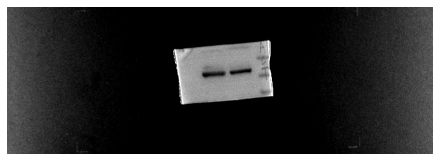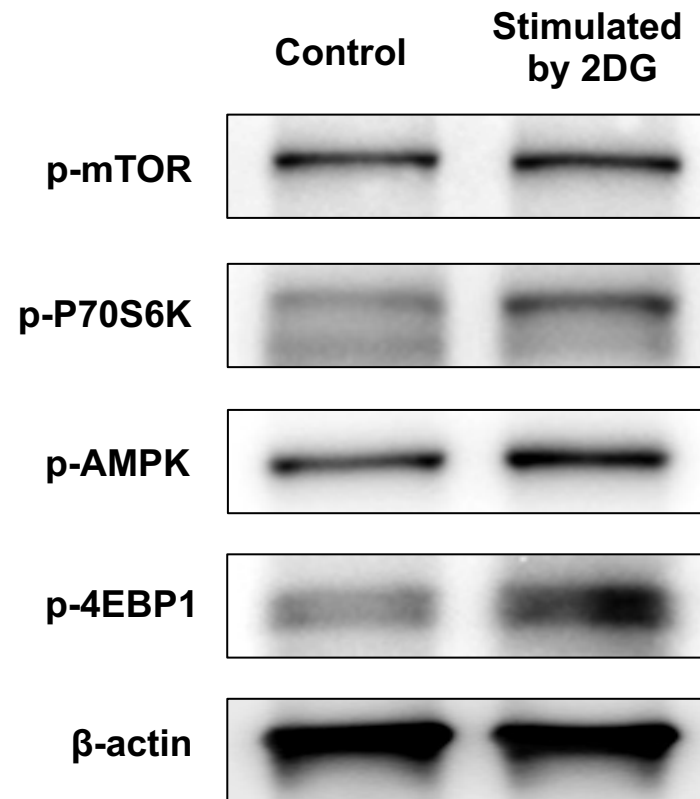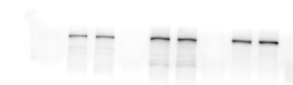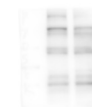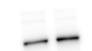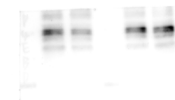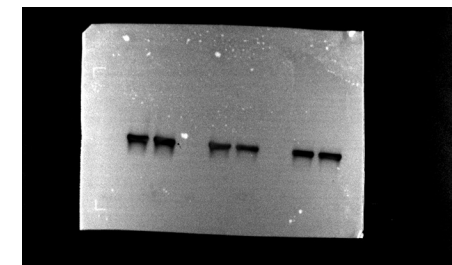

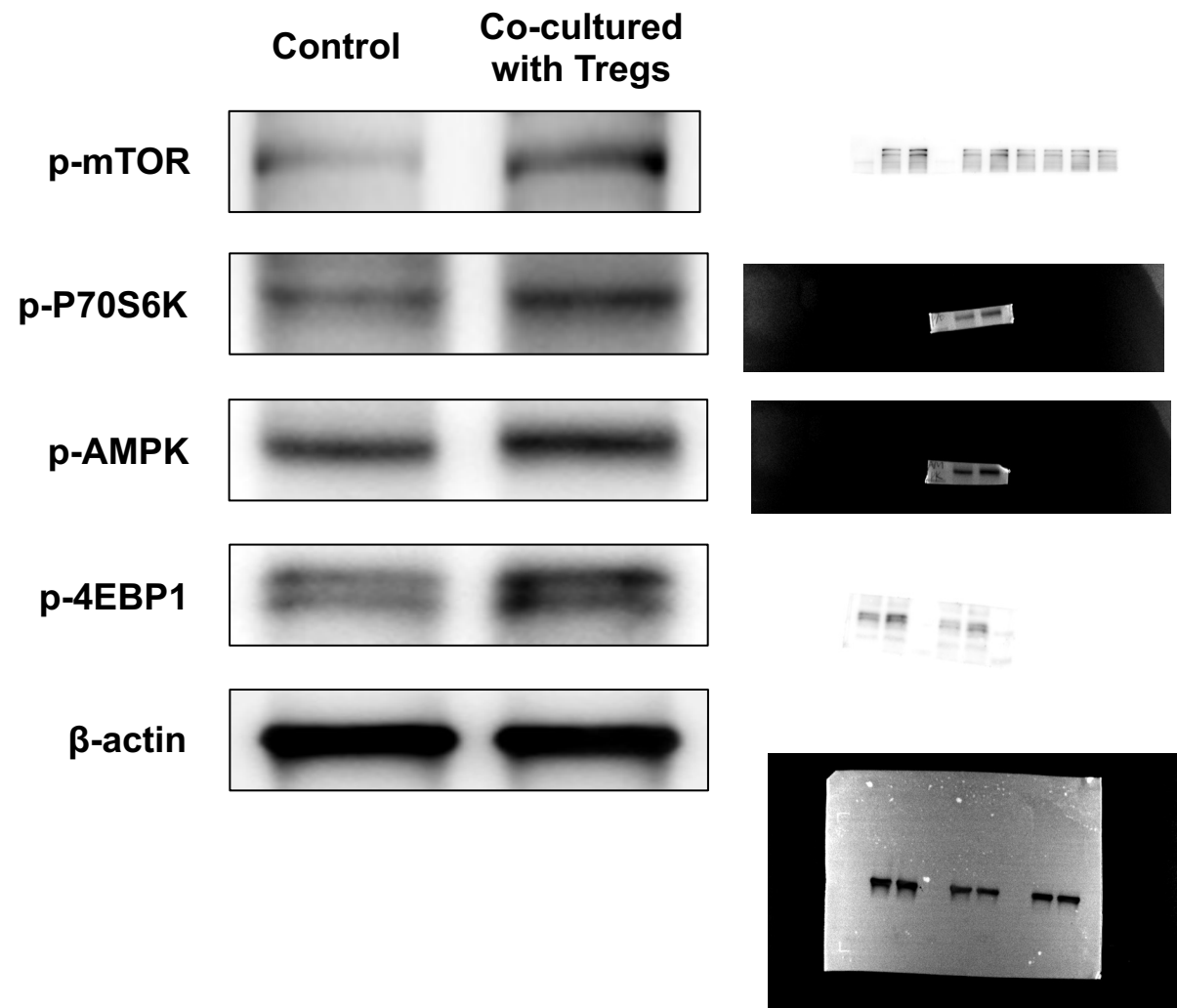

Low glucose      Stimulated by NaLA

p-mTOR

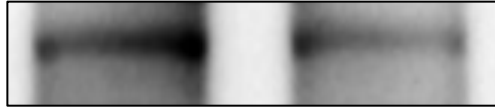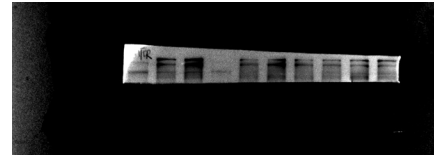

p-P70S6K

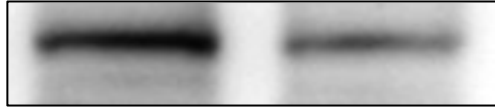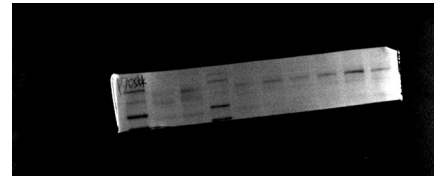

p-AMPK

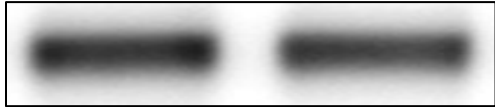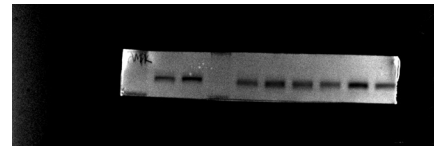

p-4EBP1

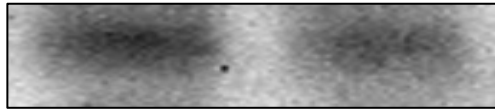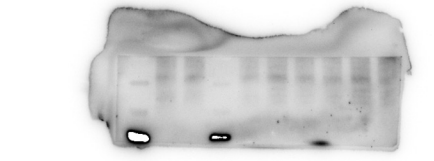

β-actin

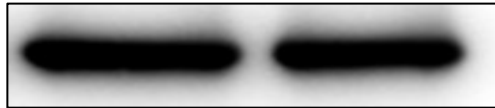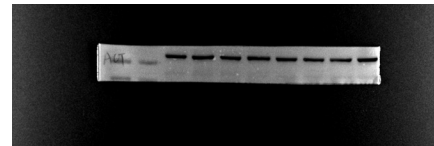

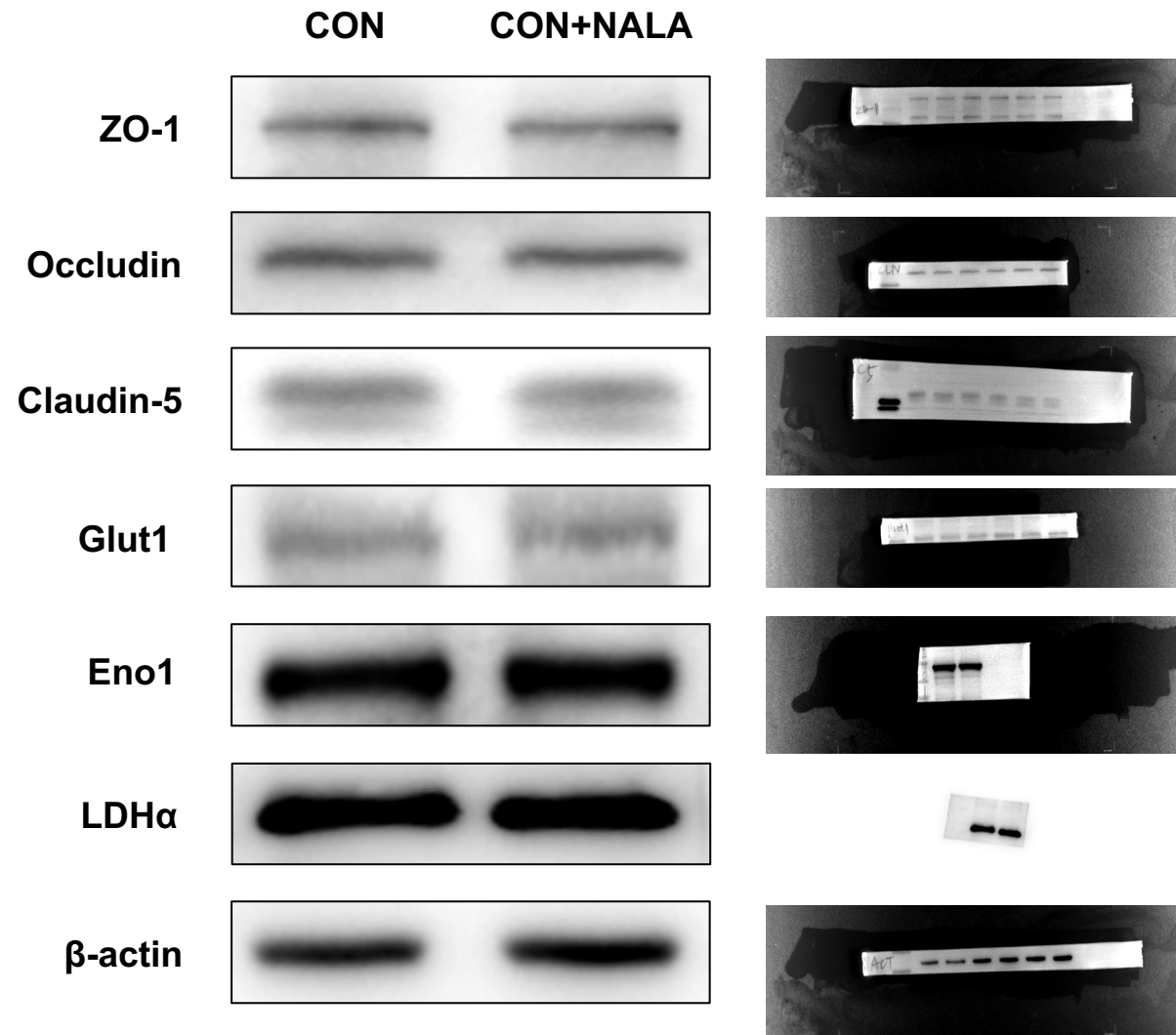

Supplement: Supplementary file 6 — Additional file 6. [file 12916_2024_3413_MOESM6_ESM.pdf]
